# Doubly Robust Estimation and Semiparametric Efficiency in Generalized Partially Linear Models with Missing Outcomes

**DOI:** 10.3390/stats7030056

**Published:** 2024-08-31

**Authors:** Lu Wang, Zhongzhe Ouyang, Xihong Lin

**Affiliations:** 1 Department of Biostatistics, University of Michigan, Ann Arbor, MI 48109, USA; 2 Department of Biostatistics, Harvard School of Public Health, Boston, MA 02115, USA

**Keywords:** asymptotics, augmented inverse probability weighting, kernel smoothing, missing data at random, profile-kernel estimating equation, semiparametric efficiency

## Abstract

We investigate a semiparametric generalized partially linear regression model that accommodates missing outcomes, with some covariates modeled parametrically and others nonparametrically. We propose a class of augmented inverse probability weighted (AIPW) kernel–profile estimating equations. The nonparametric component is estimated using AIPW kernel estimating equations, while parametric regression coefficients are estimated using AIPW profile estimating equations. We demonstrate the doubly robust nature of the AIPW estimators for both nonparametric and parametric components. Specifically, these estimators remain consistent if either the assumed model for the probability of missing data or that for the conditional mean of the outcome, given covariates and auxiliary variables, is correctly specified, though not necessarily both simultaneously. Additionally, the AIPW profile estimator for parametric regression coefficients is consistent and asymptotically normal under the semiparametric model defined by the generalized partially linear model on complete data, assuming that the missing data mechanism is missing at random. When both working models are correctly specified, this estimator achieves semiparametric efficiency, with its asymptotic variance reaching the efficiency bound. We validate our approach through simulations to assess the finite sample performance of the proposed estimators and apply the method to a study that investigates risk factors associated with myocardial ischemia.

## 1. Introduction

Generalized partially linear models,

(1) $$E(Y|𝒳,Z)=\mu \left\{{𝒳}^{T}\beta +\theta (Z)\right\}$$

where μ(⋅) is a known monotonic link function (McCullagh and Nelder 1989 [1]), Y is an outcome of interest, 𝒳 is a p×1 vector of primary covariates, Z is an additional scalar covariate, β is an unknown parameter vector of dimension p, and θ(⋅) is an unknown smooth function, have been extensively studied without missing data (Severini and Staniswalis 1994 [2]; Hastie and Tibshirani 1990 [3]; Fan, et al. 1995 [4]; Carroll, et al. 1997 [5]; Lin and Carroll 2001a [6], 2001b [7]; Muller 2001 [8]; Hu and Cui 2010 [9]; Rahman, et al. 2020 [10]). In model (1), ${𝒳}^{T}\beta$ summarizes the dependence of the outcome mean on covariates 𝒳 of interest whereas the unknown smooth function θ(⋅) allows for model flexibility for the dependence on a secondary covariate Z. Our contribution in this paper is to study the estimation of β and θ(⋅) and the asymptotics when the outcome Y is missing at random (MAR), i.e., missingness depends on observed data (Little and Rubin 2002 [11]) while some additional auxiliary variables and information exist (Chu and Halloran 2004 [12]).

Our work is motivated by the investigation of risk factors for myocardial ischemia (reduced blood flow due to obstruction in the vessels) from data collected at the radiology clinic of a nuclear imaging group. The standard technique for screening myocardial ischemia at the time of data collection was dual-isotope myocardial perfusion single-photon emission computed tomography (SPECT), whose use was not only expensive but also involved ingestion of radioactive tracing material. Because of this, only a subset of the subjects who attended the radiology clinic were actually referred to have the SPECT test performed. Instead, all subjects attending the clinic were screened with electron beam computed tomography (EBCT). This device is routinely used to measure the degree of calcification in the arteries (Braun, et al. 1996 [13]). Doctors decided whether or not to refer a subject to the SPECT test based on the information available, including the results of the EBCT test. In our investigation, we wish to make inferences about the parameter β in the logistic partially linear regression model (1), i.e., with $\mu (u)=\{1+\text{exp}(-u){\}}^{-1}$, when Y is a binary indicator of a positive SPECT test, Z is age, and 𝒳 variables include gender, smoking status, blood pressure status (high/low), cholesterol status (high/low), and the presence of chest pain, under the assumption that whether to refer a patient to the SPECT test depends only on the recorded covariates and the EBCT test. Consequently, the missingness of the outcome Y is MAR in this study.

The literature is vast on inference on regression coefficients β in parametric generalized linear models of the form $E(Y|𝒳)=\mu \left({𝒳}^{T}\beta \right)$ when outcomes are missing at random. Both likelihood-based approaches (Little 1982 [14], 1995 [15]; Little and Rubin 2002 [11]) and estimating equation-based approaches (Robins and Rotnitzky 1995 [16]; Robins et al. 1995 [17]) have been extensively studied. Inference on the nonparametric function θ(⋅) in generalized nonparametric models E(Y∣Z)=μ{θ(Z)} with outcomes missing at random has also been studied in the literature (e.g., Wang et al. 1998 [18]; Chen et al. 2006 [19]; Wang et al. 2010 [20]; and Kennedy et al. 2017 [21]). Our primary interest in this paper lies in estimating the finite dimensional parameter vector β while treating the infinite dimensional parameter θ(⋅) as a nuisance parameter under semiparametric model (1) in the presence of missing outcomes. Liang et al. (2004) [22], Liang (2008) [23], and Wang (2009) [24] considered semiparametric models in the presence of missing covariates. Wang et al. (2004) [25] and Wang and Sun (2007) [26] considered imputation and weighted estimators in partially linear models for Gaussian outcomes when outcomes are missing at random. Liang et al. (2007) [27] also extended the work to a scenario when covariates are measured with error. Chen and Keilegom (2013) [28] proposed an imputation method for semiparametric models, and Kennedy et al. (2017) [21] proposed a kernel-smoothing method for estimating continuous treatment effects, but all these authors do not allow for auxiliary covariates. To the best of our knowledge, there is no existing literature on the semiparametric efficiency bound and semiparametric efficient estimators in generalized semiparametric regression models (1) for both continuous and discrete outcomes when outcomes are missing at random in the presence of auxiliary covariates. This paper aims to fill this gap.

Specifically, this paper makes the following three major contributions and provides a comprehensive investigation of inference in the generalized semiparametric regression model (1) when outcomes are missing at random: (i) Unlike previous authors, we allow for the possibility that some auxiliary covariate(s) 𝒰 are available. For example, in the analysis of myocardial ischemia data, 𝒰 is the EBCT test result. The auxiliary covariates are not of primary interest in the sense that we are concerned with the estimation of E(Y∣𝒳,Z) rather than E(Y∣𝒳,Z,𝒰). They allow for a weaker modeling assumption that the missingness is assumed to be independent of outcomes, conditional on the auxiliary covariates. They can also help improve the efficiency in estimation of both θ(⋅) and β. (ii) We derive the explicit form of a semiparametric efficiency score and efficiency bound in generalized partially linear models in the presence of auxiliary covariates when outcomes are missing at random. (iii) We propose a locally semiparametric efficient estimator of β in model (1) that reaches the semiparametric efficiency bound when Y is missing at random. Specifically, we propose augmented inverse probability weighted (AIPW) kernel–profile estimating equations where for a given β, the nonparametric function θ(⋅) is estimated using the AIPW kernel estimating equation and the parametric regression coefficient β is estimated using the AIPW profile estimating equation of β given in Equation (6). The joint estimation of θ(z) and β proceeds by iteratively solving the two sets of equations. Construction of the proposed estimators requires the specification of a parametric model for the missing data mechanism and a parametric model for E(Y∣𝒳,Z,𝒰). Yet, consistency of the proposed estimators of θ(⋅) and β requires that one of these models is correctly specified but not necessarily both, that is, the proposed estimators of both β and θ(⋅) are doubly robust (Robins and Rotnitzky, 1995 [16]; Robins et al. 1994 [29], 1995 [17]; Rotnitzky et al. 1998 [30]; and Bang and Robins 2005 [31]). In addition, the proposed estimator of β achieves the semiparametric efficiency bound when both models are correctly specified.

The rest of this paper is organized as follows. Section 2 formalizes the inferential problem. Section 3 delineates our proposed method for constructing estimators for both β and θ(⋅). In Section 4, we delve into the study of asymptotic efficiency concerning the estimation of β, presenting the derived semiparametric efficient score and efficiency bound. Section 5 examines the asymptotic properties of the proposed estimators for both β and θ(⋅), emphasizing the local semiparametric efficiency of our proposed estimator for β. Subsequently, Section 6 conducts a simulation study to evaluate the finite sample performance of the proposed methods, while Section 7 applies these methods to analyze data stemming from the myocardial ischemia study. Finally, we offer concluding remarks in Section 8.

## 2. A Formalization of the Inferential Problem

Suppose we would ideally like to measure variables (Y,𝒳,Z) on a random sample of n subjects from a population of interest, where the variables follow model (1) with a known monotonic link function μ(⋅) that has a continuous first derivative, $\beta \in \underset{\_}{\beta }$, an open set in ${ℛ}^{p}$, and an unknown smooth function θ(⋅). For example, in the myocardial ischemia study, we include age as a nonparametric predictor due to the potential nonlinear effect of aging on the risk of myocardial ischemia, and we model all other covariates parametrically to avoid the curse of dimensionality. In this paper, we discuss the estimation of β and θ(⋅) in settings where Y is only observed on a subsample, but (𝒳,Z) and additional auxiliary variables 𝒰 are always observed under the assumption that Y is missing at random (Little and Rubin 2002 [11]), i.e.,

(2) Pr(R=1∣𝒳,Z,𝒰,Y)=Pr(R=1∣𝒳,Z,𝒰),

where R=1 if Y is observed and R=0 otherwise. Under assumption (2), missingness of the outcome Y may depend on 𝒳, Z, and 𝒰 but is independent of Y given (𝒳,Z,𝒰). In the myocardial ischemia study described in the Introduction, assumption (2) would hold if the variables 𝒳, Z and the EBCT test result 𝒰 were the only correlates of the SPECT test result that were used by doctors to decide the SPECT test referral status.

In our context, it is worth noting that the variables 𝒰 are not our primary focus; that is, our concern lies in estimating E(Y∣𝒳,Z) rather than E(Y∣𝒳,Z,𝒰). However, these auxiliary variables may be necessary to ensure that the missingness is conditionally independent of the outcome Y. For instance, in the myocardial ischemia study, our primary interest does not lie in the relationship between EBCT and SPECT test results but rather in understanding the risk of a positive SPECT test result, indicative of myocardial ischemia, in relation to Z and 𝒳—factors like age, gender, smoking, and other health indicators. Nevertheless, if the referral to the SPECT exam within the strata of 𝒳 and Z were influenced by the EBCT test values, then (2) would fail if 𝒰 were omitted from both sides of the equation, because the EBCT and SPECT test results exhibit correlation within the strata defined by (𝒳,Z).

We consider the estimation of β and θ(⋅) when Y is either missing by happenstance, where $\pi_{0}(𝒳,Z,𝒰)\equiv Pr(R=1|𝒳,Z,𝒰)$, which is sometimes abbreviated as $\pi_{0}$ when no confusion exists, is an unknown function of (𝒳,Z,𝒰) consequently, as in the case of the myocardial ischemia study, or missing by design, where $\pi_{0}$ is a known function as it is the case in a designed two-stage study (Pepe, 1992 [32]; Reilly and Pepe, 1995 [33]). In the latter case, (𝒳,Z,𝒰) are measured across the entire sample in the initial stage, followed by the selection of a subsample in the subsequent stage, with selection probabilities contingent on the data from the first stage, and Y is measured within this subsample.

## 3. The Estimation Procedure

### 3.1. The AIPW Kernel–Profile Estimating Equations

Our estimating procedure is based on augmented inverse probability weighted (AIPW) kernel–profile estimating equations, where θ(⋅) is estimated using AIPW kernel estimating equations (Wang et al. 2010 [20]) and β is estimated using profile-type AIPW estimating equations. An AIPW kernel- or profile-type estimating function is constructed as the sum of an inverse probability weighted (IPW) estimating function, corresponding to a kernel- or profile-type, and a specific augmentation term, with weights equal to either the inverse of $\pi_{0}$ if $\pi_{0}$ is known, as in designed two-stage studies, or the inverse of $\hat{\pi }\equiv \pi (𝒳,Z,𝒰;\hat{\tau })$ if $\pi_{0}$ is unknown, as when Y is missing by happenstance, where $\hat{\tau }$ is the maximum likelihood estimator of $\tau \in R^{k}$ under a postulated parametric model,

(3) Pr(R=1∣𝒳,Z,𝒰)=π(𝒳,Z,𝒰;τ),

and π(𝒳,Z,𝒰;τ) is a known smooth function subject to τ. For example, $\pi (𝒳,Z,𝒰;\tau )=\text{expit}\left\{\tau_{0}+\tau_{1}^{T}𝒳+\tau_{2}Z+\tau_{3}^{T}𝒰\right\}$, where $\tau ={\left(\tau_{0},\tau_{1}^{T},\tau_{2},\tau_{3}^{T}\right)}^{T}$ and expit(x)=exp(x)/{1+exp(x)}. A special case when there are no augmentation terms is referred to as the IPW kernel–profile estimating equations, which are similar to those described in Carroll et al. (1997) [5] and Wang et al. (2005) [34], based on units with Y observed but with each contributing unit weighted by the inverse of its selection probability, if known, or an estimate of it otherwise. The IPW estimators are easier to compute than the AIPW estimator. However, as we shall see, the IPW estimators are generally not efficient nor doubly robust.

To construct an AIPW kernel–profile estimator $\left\{{\hat{\theta }}_{\text{AIPW}}(\cdot ),{\hat{\beta }}_{\text{AIPW}}\right\}$, we initially input a user-specified p×1 function δ(𝒳,Z,𝒰) and postulate a working model

(4) $$var\left(\epsilon_{\delta }^{*}|𝒳,Z\right)=V(𝒳,Z;\zeta )$$

for the conditional variance of

$$\epsilon_{\delta }^{*}\equiv \frac{R}{\pi_{0}}\epsilon -\left(\frac{R}{\pi_{0}}-1\right)\times \left[\delta (𝒳,Z,𝒰)-\mu \left\{{𝒳}^{T}\beta +\theta (Z)\right\}\right],$$

with $\epsilon \equiv Y-\mu \left\{{𝒳}^{T}\beta_{0}+\theta_{0}(Z)\right\}$, $\beta_{0}$ and $\theta_{0}(\cdot )$ as the true values of β and θ(⋅), V(𝒳,Z;⋅) as a known smooth function, and $\zeta \in R^{r}$ as an unknown finite dimensional parameter vector.

For conciseness, here we describe AIPW local linear kernel–profile estimating equations. Extensions to weighted local polynomial estimating equations are straightforward. In what follows, $K_{h}(s)=h^{-1}K(s/h)$, where K(⋅) is a mean-zero density function, $\alpha ={\left(\alpha_{0},\alpha_{1}\right)}^{T}$, and for any scalar u we let $𝒢(u)=(1,u{)}^{T}$. We describe the algorithm for the case in which $\pi_{0}$ is unknown. For the case where $\pi_{0}$ is known, the algorithm differs only in that all instances of $\hat{\pi }$ are replaced by $\pi_{0}$.

We start the algorithm with an initial estimator $\left\{\overset{ˇ}{\theta }\left(Z_{1}\right),\ldots ,\overset{ˇ}{\theta }\left(Z_{n}\right),\overset{ˇ}{\beta }\right\}$, satisfying $n^{\left(1/2\right)}(\overset{ˇ}{\beta }-\beta )=O_{p}(1)$. Such an initial estimator can be obtained by modifying the estimators described in Carroll et al. (1997) [5] based on the completed units (those with Y observed) weighted by the inverse of their selection probability, if known, or an estimate of it otherwise, with $V_{i}$ replaced by $V\left({𝒳}_{i},Z_{i};\tilde{\zeta }\right)$ for any user-specified fixed constant $\tilde{\zeta }$. To compute $\hat{\zeta }$, we calculate $S_{i}={\left[R_{i}{\hat{\pi }}_{i}^{-1}\left(Y_{i}-Q_{i}\right)-\left(R_{i}{\hat{\pi }}_{i}^{-1}-1\right)\left\{\delta \left({𝒳}_{i},Z_{i},{𝒰}_{i}\right)-Q_{i}\right\}\right]}^{2}$ where $Q_{i}=\mu \left\{{𝒳}_{i}^{T}\overset{ˇ}{\beta }+\overset{ˇ}{\theta }\left(Z_{i}\right)\right\}$, i=1,…,n. The estimator $\hat{\zeta }$ is obtained by nonlinear least squares regression of $S_{i}$ on ${𝒳}_{i}$,$Z_{i}$ under the model $E\left(S_{i}|{𝒳}_{i},Z_{i}\right)=V\left({𝒳}_{i},Z_{i};\zeta \right)$. Then we iterate the following two steps until convergence:

1. For the fixed β and any given z, we calculate $\hat{\theta }(z,\beta )$ using an AIPW kernel estimating equation similar to Wang et al. (2010) [20]. Specifically, $\hat{\theta }(z,\beta )$ is defined as ${\hat{\alpha }}_{0}(\beta )$, the first component of the vector $\hat{\alpha }(\beta )={\left\{{\hat{\alpha }}_{0}(\beta ),{\hat{\alpha }}_{1}(\beta )\right\}}^{T}$, solving the following AIPW kernel estimating equations in α,
(5) $$\sum_{i=1}^{n}K_{h}\left(Z_{i}-z\right)\mu_{i,z}^{\left(1\right)}(\alpha )V_{i}^{-1}𝒢\left(Z_{i}-z\right)\left[\frac{R_{i}}{{\hat{\pi }}_{i}}\cdot \left\{Y_{i}-\mu_{i,z}(\alpha )\right\}\right.\;\left.-\left(\frac{R_{i}}{{\hat{\pi }}_{i}}-1\right)\cdot \left\{\delta \left({𝒳}_{i},Z_{i},{𝒰}_{i}\right)-\mu_{i,z}(\alpha )\right\}\right]=0,$$
  where, to simplify notation, $\mu_{i,z}(\alpha )=\mu \left\{{𝒳}_{i}^{T}\beta +𝒢{\left(Z_{i}-z\right)}^{T}\alpha \right\}$, $\mu_{i,z}^{\left(1\right)}(\alpha )$ is the first derivative of μ(r) with respect to r when r is evaluated at ${𝒳}_{i}^{T}\beta +𝒢{\left(Z_{i}-z\right)}^{T}\alpha$ and $V_{i}=V\left({𝒳}_{i},Z_{i};\hat{\zeta }\right)$, with $\hat{\zeta }$ defined above.
2. We compute $\hat{\beta }$ by solving the following AIPW profile estimating equation in β,
(6) $$\sum_{i=1}^{n}{\tilde{\mu }}_{i}^{\left(1\right)}(\beta )V_{i}^{-1}\left\{{𝒳}_{i}+\frac{\partial \hat{\theta }\left(Z_{i},\beta \right)}{\partial \beta }\right\}\left[\frac{R_{i}}{{\hat{\pi }}_{i}}\cdot \left\{Y_{i}-{\tilde{\mu }}_{i}\left(\beta \right)\right\}\;\left.-\left(\frac{R_{i}}{{\hat{\pi }}_{i}}-1\right)\cdot \left\{\delta \left({𝒳}_{i},Z_{i},{𝒰}_{i}\right)-{\tilde{\mu }}_{i}\left(\beta \right)\right\}\right]=0,\right.$$
  where ${\tilde{\mu }}_{i}(\beta )=\mu \left\{{𝒳}_{i}^{T}\beta +\hat{\theta }\left(Z_{i},\beta \right)\right\},{\tilde{\mu }}_{i}^{\left(1\right)}(\beta )$ is the first derivative of μ(r) with respect to r when r is evaluated at ${𝒳}_{i}^{T}\beta +\hat{\theta }\left(Z_{i},\beta \right)$.

At convergence, we obtain ${\hat{\beta }}_{\text{AIPW}}$ and ${\hat{\theta }}_{\text{AIPW}}(\cdot )=\hat{\theta }\left(\cdot ,{\hat{\beta }}_{\text{AIPW}}\right)$. When the link function μ(⋅) is the identity and $V_{i}\text{s}$ are constants, both ${\hat{\beta }}_{\text{AIPW}}$ and ${\hat{\theta }}_{\text{AIPW}}(z)$ have a closed form and are linear functions of Y.

We can similarly define the simpler-to-compute nonaugmented inverse probability weighted (IPW) estimators ${\hat{\beta }}_{IPW}$ and ${\hat{\theta }}_{IPW}(z)$ of β and θ(⋅), which are the output of the iterative two-step procedure described above with Equations (5) and (6) replaced by

(7) $$\sum_{i=1}^{n}\frac{R_{i}}{{\hat{\pi }}_{i}}K_{h}\left(Z_{i}-z\right)\mu_{i,z}^{\left(1\right)}(\alpha )V_{i}^{-1}𝒢\left(Z_{i}-z\right)\left\{Y_{i}-\mu_{i,z}(\alpha )\right\}=0$$

and

(8) $$\sum_{i=1}^{n}\frac{R_{i}}{{\hat{\pi }}_{i}}{\tilde{\mu }}_{i}^{\left(1\right)}(\beta )V_{i}^{-1}\left\{{𝒳}_{i}+\frac{\partial \hat{\theta }\left(Z_{i},\beta \right)}{\partial \beta }\right\}\left\{Y_{i}-{\tilde{\mu }}_{i}(\beta )\right\}=0$$

with $\hat{\zeta }$ obtained by regressing ${\tilde{S}}_{i}\equiv {\left[R_{i}{\hat{\pi }}_{i}^{-1}\cdot \left(Y_{i}-\mu \left\{{𝒳}_{i}^{T}\beta +\overset{ˇ}{\theta }\left(Z_{i}\right)\right\}\right)\right]}^{2}$ on ${𝒳}_{i}$ and $Z_{i}$, i=1,…,n, under a model $V\left({𝒳}_{i},Z_{i};\zeta \right)$ for $E\left({\tilde{S}}_{i}|{𝒳}_{i},Z_{i}\right)$.

Choosing an appropriate bandwidth parameter h is important when estimating θ(⋅). We generalize the empirical bias bandwidth selection (EBBS) method of Ruppert (1997) [35] to derive a data-driven bandwidth selection approach in practice; for details, refer to Section 4.3 in Wang et al. 2010 [20]. In Section 5, we derive the asymptotic properties of both $\left\{{\hat{\beta }}_{\text{AIPW}},{\hat{\theta }}_{\text{AIPW}}(z)\right\}$ and $\left\{{\hat{\beta }}_{IPW},{\hat{\theta }}_{IPW}(z)\right\}$.

### 3.2. Doubly Robust, Locally Efficient Estimation

If $\pi_{0}$ is unknown, the consistency of the estimators ${\hat{\beta }}_{\text{AIPW}}$ and ${\hat{\theta }}_{\text{AIPW}}(z)$ requires model (3) for the selection probabilities to be correctly specified. This can be relaxed by a slight modification to the preceding algorithm. Specifically, consider new estimators ${\hat{\beta }}_{DR}$ and ${\hat{\theta }}_{DR}(z)$ obtained by replacing δ(𝒳,Z,𝒰) with $\delta (𝒳,Z,𝒰;\hat{\eta })$, where $\hat{\eta }$ is the possibly weighted least squares estimator of η under the model

(9) E(Y∣𝒳,Z,𝒰)=δ(𝒳,Z,𝒰;η).

In Section 5, we show that under regularity conditions, ${\hat{\beta }}_{DR}$ and ${\hat{\theta }}_{DR}(z)$ are consistent for β and θ(z) provided either model (3) or model (9) is correctly specified but not necessarily both. This property is often referred to as double-robustness.

In addition to more protection against model misspecification, the estimators ${\hat{\beta }}_{DR}$ and ${\hat{\theta }}_{DR}(z)$ have attractive asymptotic efficiency properties. Specifically, if model (3) and model (9) are both correctly specified, then ${\hat{\theta }}_{DR}(z)$ has asymptotic variance that is equal to the smallest possible asymptotic variance of ${\hat{\theta }}_{\text{AIPW}}(z)$, as δ(𝒳,Z,𝒰) ranges over all possible functions. In addition, ${\hat{\beta }}_{DR}$ is locally semiparametric efficient under the semiparametric model defined by the restrictions (1)–(3) of the submodel defined by the additional restrictions (4) and (9). That is, under regularity conditions, ${\hat{\beta }}_{DR}$ is consistent and asymptotically normal for β when the selection probability satisfies (3); if in addition, the true data generating process satisfies the working models (4) and (9), then its limiting distribution has variance equal to the semiparametric variance bound for regular estimators of β in the semiparametric model defined by restrictions (1)–(3). We explicitly demonstrate these properties in Sections 4 and 5.

## 4. Semiparametric Efficiency Theory for Estimation of β

The semiparametric variance bound for estimators of a finite-dimensional parameter β within an arbitrary semiparametric model serves as the counterpart to the Cramer–Rao bound in parametric models. This bound is defined as the supremum of the Cramer–Rao bounds for β across all regular parametric submodels (Begun et al., 1983 [36]; Newey, 1990 [37]; Bickel et al., 1993 [38]). Analogous to its parametric counterpart, this bound offers a benchmark against which the efficiency of estimators of β that are consistent and asymptotically normal—more precisely, regular and asymptotically linear (RAL) under the semiparametric model— can be assessed. Notably, the semiparametric bound emerges as the reciprocal of the variance of the semiparametric efficient score for β.

In this section, we elucidate the semiparametric efficient score and semiparametric variance bound for β within the semiparametric model 𝒜, governing the law $F_{O}$ of the observed data O=(R,RY,𝒳,Z,𝒰) defined by restriction (1) on the full data and the MAR restriction (2) on the missing data mechanism. To achieve this, we draw upon the general theory established by Ibragimov and Hasminskii (1981) [39], Robins and Rotnitzky (1992) [40], Robins et al. (1994) [29], and Rotnitzky and Robins (1997) [41], and discussed in van der Laan and Robins (2003) [42] and Tsiatis (2006) [43], among others. The derivation requires the characterization of $\Lambda_{\text{nuis}}^{⊥}$, the orthocomplement of the nuisance tangent space, i.e., of the closed linear span of nuisance scores under model 𝒜, in the Hilbert space $L_{2}\left(F_{O}\right)$ of mean-zero, finite variance scalar functions T=t(O) with covariance inner product. We characterize $\Lambda_{\text{nuis}}^{⊥}$ by $\Lambda_{\text{nuis}}^{⊥,\text{full}}$, the orthocomplement to the nuisance tangent space for β under the semiparametric model ${𝒜}_{\text{full}}$ for the law $F_{W}$ of the full data W=(Y,𝒳,Z,𝒰) defined by restriction (1) in the Hilbert space $L_{2}\left(F_{W}\right)$. This is so since, as shown in Robins and Rotnitzky (1992) [40],

(10) $$\Lambda_{\text{nuis}}^{⊥}=\left\{\frac{R}{\pi_{0}}Q-\left(\frac{R}{\pi_{0}}-1\right)E(Q|R=1,𝒳,Z,𝒰):Q\in \Lambda_{\text{nuis}}^{⊥,\text{full}}\right\}.$$

In Supplementary Materials S1, we show that $\Lambda_{\text{nuis}}^{⊥,\text{full}}$ is composed of all finite variance functions of the form b(𝒳,Z)ϵ, where ϵ is defined in Section 3.1, with b(𝒳,Z) satisfying

(11) $$E\left[b(𝒳,Z)\mu^{\left(1\right)}\left\{{𝒳}^{T}\beta_{0}+\theta_{0}(Z)\right\}|Z\right]=0,$$

where $\mu^{\left(1\right)}\{\cdot \}$ is the first derivative of μ(⋅). Robins et al. (1994) [29] derived $\Lambda_{\text{nuis}}^{⊥,\text{full}}$ for μ(u)=u, Bickel et al. (Sec 4.3, 1993) [38] (Sec 4.3, 1993) for $\mu (u)=\{1+\text{exp}(-u){\}}^{-1}$, and Robins and Rotnitzky (2001) [44] for μ(u)=exp(u).

According to Bickel et al. 1993 [38], the semiparametric efficient score ${𝒮}_{\text{eff}}$ for β in model 𝒜 at $F_{O}$ is a p×1 vector whose elements belong to $\Lambda_{\text{nuis}}^{⊥}$. Consequently, in view of (10), ${𝒮}_{\text{eff}}$ must be equal to $b_{\text{eff}}(𝒳,Z)\epsilon^{*}$ for some p×1 function $b_{\text{eff}}(𝒳,Z)$, whose elements satisfy (11), and with

(12) $$\epsilon^{*}=\frac{R}{\pi_{0}}\epsilon -\left(\frac{R}{\pi_{0}}-1\right)E\left(\epsilon |𝒳,Z,𝒰\right).$$

In S.1, we show that $b_{\text{eff}}(𝒳,Z)=\sigma^{-2}(𝒳,Z)\mu^{\left(1\right)}\left\{{𝒳}^{T}\beta_{0}+\theta_{0}(Z)\right\}\left(𝒳-\varphi_{\text{eff}}\right)$, where $\sigma^{2}(𝒳,Z)=var\left(\epsilon^{*}|𝒳,Z\right)$ and

$$\varphi_{eff}=\frac{E\left\{{\left[\mu^{\left(1\right)}\left\{{𝒳}^{T}\beta_{0}+\theta_{0}(Z)\right\}\right]}^{2}\sigma^{-2}(𝒳,Z)𝒳|Z\right\}}{E\left\{{\left[\mu^{\left(1\right)}\left\{{𝒳}^{T}\beta_{0}+\theta_{0}(Z)\right\}\right]}^{2}\sigma^{-2}(𝒳,Z)|Z\right\}}.$$

It then follows that the semiparametric variance bound ${𝒱}_{eff}=E{\left({𝒮}_{eff}{𝒮}_{eff}^{T}\right)}^{-1}$ for β in the observed data model is equal to

(13) $${𝒱}_{eff}={\left\{E\left[\sigma^{-2}(𝒳,Z){\left[\mu^{\left(1\right)}\left\{{𝒳}^{T}\beta_{0}+\theta_{0}(Z)\right\}\right]}^{2}\left(𝒳-\varphi_{eff}\right){\left(𝒳-\varphi_{eff}\right)}^{T}\right]\right\}}^{-1}.$$

In fact, the semiparametric efficient score and efficiency bound given above are also the ones corresponding to a model which additionally imposes model (3) on the selection probabilities. This is so because under MAR, the likelihood factorizes into a part that depends on the selection probabilities and another part that depends on β and θ(⋅).

## 5. Asymptotic Properties

In this section, we investigate the asymptotic properties of the AIPW and IPW profile–kernel estimators. For conciseness, we only present our asymptotic results for the local linear kernel–profile estimators. The results can be extended to local polynomial regression. Here and throughout, we make the following assumptions: (I) n→∞, h→0, and nh→∞; (II) z is in the interior of the support of Z; (III) for some constant c, Pr(R=1∣𝒳,Z,𝒰)>c>0 with probability 1 in a neighborhood of Z=z; and (IV) the regularity conditions stated at the beginning of the Supplementary Materials hold.

Under the aforementioned assumptions and MAR, both the IPW and AIPW kernel estimators are consistent for θ(z) provided that the estimating equations use either the true selection probabilities or $\sqrt{n}$–consistent estimates under a correctly specified model (3). Furthermore, the AIPW estimator of θ(z) that uses $\delta \left({𝒳}_{i},Z_{i},{𝒰}_{i}\right)=\delta \left({𝒳}_{i},Z_{i},{𝒰}_{i};\hat{\eta }\right)$, as defined before, remains consistent if model (9) for the conditional mean of Y given 𝒳, Z, and 𝒰 is correctly specified even if model (3) for the selection probability is misspecified. These are similar to the findings in Wang et al. (2010) [20], but given that this paper's model is different with ${𝒳}^{T}\beta$ additionally compared to theirs, some of the expressions are slightly different. So we summarize the asymptotic distributions of ${\hat{\theta }}_{IPW}(z)$ and ${\hat{\theta }}_{\text{AIPW}}(z)$ briefly in the following Theorems 1 and 2.

**Theorem 1.** *Suppose that* Equation (5) *uses (a)* ${\hat{\pi }}_{i}$ *that is computed under model* (3) *or is replaced by fixed probabilities* $\pi_{i}^{*}$, *and (b) a fixed function* $\delta^{*}(𝒳,Z,𝒰)$ *or* $\delta (𝒳,Z,𝒰)=\delta (𝒳,Z,𝒰;\hat{\eta })$, *where* $\hat{\eta }$ *is a* $\sqrt{n}$-*consistent estimator of* η *under model* (9). *Suppose the MAR assumption* (2) *and assumptions I)-IV) above hold and further that either of the following hold: (i) model* (3) *is correct or, if* $\pi_{i}^{*}$ *is used*, $\pi_{i}^{*}=\pi_{i0}$ *for all* i, *or (ii)* $\delta^{*}(𝒳,Z,𝒰)=E(Y|𝒳,Z,𝒰)$ *or if* $\delta (𝒳,Z,𝒰;\hat{\eta })$ *is used, model* (9) *is correctly specified. Then:*

1. *There exists a sequence of solutions* ${\hat{\theta }}_{\text{AIPW}}\left(z;\beta_{0}\right)$ *of* (5) *such that*
(14) $$\sqrt{nh}\left\{{\hat{\theta }}_{\text{AIPW}}\left(z;\beta_{0}\right)-\theta (z)-\frac{1}{2}h^{2}\theta^{\prime \prime }(z)c_{2}(K)+o\left(h^{2}\right)\right\}\to N\left\{0,\Sigma_{\theta ,\text{AIPW}}(z)\right\},$$
  *where*
(15) $$\Sigma_{\theta ,\text{AIPW}}(z)=\frac{c_{0}\left(K^{2}\right)}{t(z)f_{Z}(z)}E\left[r(𝒳,Z)\left\{\frac{\pi_{0}\left(𝒳,Z,𝒰\right)}{{\tilde{\pi }}^{2}\left(𝒳,Z,𝒰\right)}var(Y|𝒳,Z,𝒰)+\frac{\pi_{0}\left(𝒳,Z,𝒰\right)}{\tilde{\pi }\left(𝒳,Z,𝒰\right)}\times {\left[E\left(Y|𝒳,Z,𝒰\right)-\mu \left\{\theta \left(Z\right)+{𝒳}^{T}\beta_{0}\right\}\right]}^{2}\;+\left\{\frac{\pi_{0}\left(𝒳,Z,𝒰\right)}{{\tilde{\pi }}^{2}\left(𝒳,Z,𝒰\right)}-\frac{\pi_{0}\left(𝒳,Z,𝒰\right)}{\tilde{\pi }\left(𝒳,Z,𝒰\right)}\right\}\{E\left(Y|𝒳,Z,𝒰\right)-\tilde{\delta }\left(𝒳,Z,𝒰\right){\}}^{2}\;\left.\left.+\left\{1-\frac{\pi_{0}\left(𝒳,Z,𝒰\right)}{\tilde{\pi }\left(𝒳,Z,𝒰\right)}\right\}{\left[\tilde{\delta }\left(𝒳,Z,𝒰\right)-\mu \left\{\theta \left(Z\right)+{𝒳}^{T}\beta_{0}\right\}\right]}^{2}\right\}|Z=z\right],\right.\right.$$
  $\theta^{\prime \prime }(\cdot )$ *is the second derivative of* θ(⋅), $c_{0}\left(K^{2}\right)=\int K^{2}(s)ds$, $f_{Z}(\cdot )$ *denotes the density function of* Z, $t(z)=E\left({\left[\mu^{\left(1\right)}\left\{\theta (Z)+{𝒳}^{T}\beta_{0}\right\}\right]}^{2}V^{-1}\left\{\theta (Z)+{𝒳}^{T}\beta_{0}\right\}|Z=z\right)$, $r(𝒳,Z)={\left[\mu^{\left(1\right)}\left\{\theta (Z)+{𝒳}^{T}\beta_{0}\right\}\right]}^{2}V^{-2}\left\{\theta (Z)+{𝒳}^{T}\beta_{0}\right\},c_{2}(K)=\int s^{2}K(s)ds$, $\tilde{\pi }(𝒳,Z,𝒰)$ *denotes* $\pi^{*}(𝒳,Z,𝒰)$ *if* $\pi_{i}^{*}$ *is used or the probability limit of* $\pi (𝒳,Z,𝒰;\hat{\tau })$ *if* ${\hat{\pi }}_{i}$ *is used, and* $\tilde{\delta }(𝒳,Z,𝒰)$ *denotes* $\delta^{*}(𝒳,Z,𝒰)$ *if* $\delta^{*}(𝒳,Z,𝒰)$ *is used or the probability limit of* $\delta (𝒳,Z,𝒰;\hat{\eta })$ *if* $\delta (𝒳,Z,𝒰;\hat{\eta })$ *is used*.
2. *If model* (3) *is correctly specified or if* $\pi_{i}^{*}=\pi_{i0}$ *for all* i, *then* $\tilde{\pi }(𝒳,Z,𝒰)=\pi (𝒳,Z,𝒰)$, *and* $\Sigma_{\theta ,\text{AIPW}}(z)$ *simplifies to*
$$\frac{c_{0}\left(K^{2}\right)}{t(z)f_{Z}(z)}E[r\left(𝒳,Z\right)\left\{\frac{var\left(Y|𝒳,Z,𝒰\right)}{\pi_{0}\left(𝒳,Z,𝒰\right)}+{\left[E\left(Y|𝒳,Z,𝒰\right)-\mu \left\{\theta \left(Z\right)+{𝒳}^{T}\beta_{0}\right\}\right]}^{2}\;\left.\left.+\left\{\frac{1}{\pi_{0}\left(𝒳,Z,𝒰\right)}-1\right\}[E(Y|𝒳,Z,𝒰)-\tilde{\delta }(𝒳,Z,𝒰){]}^{2}\right\}|Z=z\right]\right.,$$
  *which is minimized when* $\tilde{\delta }(𝒳,Z,𝒰)=E[Y|𝒳,Z,𝒰]$. *The corresponding* ${\hat{\theta }}_{\text{opt},\text{AIPW}}\left(z;\beta_{0}\right)$ *using either* $\delta^{*}(𝒳,Z,𝒰)=E(Y|𝒳,Z,𝒰)$ *or* $\delta (𝒳,Z,𝒰;\hat{\eta })$ *from a correctly specified model* (9) *for* E(Y∣𝒳,Z,𝒰) *has the smallest asymptotic variance among all* ${\hat{\theta }}_{\text{AIPW}}\left(z;\beta_{0}\right)$. *The asymptotic variance of* ${\hat{\theta }}_{\text{opt},\text{AIPW}}\left(z;\beta_{0}\right)$ *is equal to*
$$\frac{c_{0}\left(K^{2}\right)}{t(z)f_{Z}(z)}E\left[\left.r(𝒳,Z)\left\{\frac{var(Y|𝒳,Z,𝒰)}{\pi_{0}(𝒳,Z,𝒰)}+{\left[E(Y|𝒳,Z,𝒰)-\mu \left\{\theta (Z)+{𝒳}^{T}\beta_{0}\right\}\right]}^{2}\right\}\right|Z=z\right].$$

Part (1) of Theorem 1 formally states the important double-robustness property of ${\hat{\theta }}_{\text{AIPW}}\left(z;\beta_{0}\right)$. It stipulates that ${\hat{\theta }}_{\text{AIPW}}\left(z;\beta_{0}\right)$ is asymptotically unbiased as h→0 when n→∞ if either the model (3) for the selection probability or the model (9) for E(Y∣𝒳,Z,𝒰) is correctly specified but not necessarily both. Part (1) of Theorem 1 also states that ${\hat{\theta }}_{\text{AIPW}}\left(z;\beta_{0}\right)$ converges to $\theta \left(z;\beta_{0}\right)$ at the rate $\sqrt{nh}$, and it provides the general form of its asymptotic variance, which does not depend on the working variance V(⋅). Thus, misspecification of the working model for $var\left\{\epsilon_{\delta }^{*}(\beta ,\theta )|{𝒳}_{i},Z_{i}\right\}$ does not impact the asymptotic efficiency of ${\hat{\theta }}_{\text{AIPW}}\left(z;\beta_{0}\right)$. When model (3) for π(𝒳,Z,𝒰) is misspecified but model (9) for E(Y∣𝒳,Z,𝒰) is correctly specified, the asymptotic variance of ${\hat{\theta }}_{\text{AIPW}}\left(z;\beta_{0}\right)$ that uses $\hat{\pi }$ and $\delta (𝒳,Z,𝒰)=\delta (𝒳,Z,𝒰;\hat{\eta })$ simplifies to

$$\frac{c_{0}\left(K^{2}\right)E\left[\left.r(𝒳,Z)\left\{\frac{\pi_{0}(𝒳,Z,𝒰)var(Y|𝒳,Z,𝒰)}{{\tilde{\pi }}^{2}(𝒳,Z,𝒰)}+{\left[E(Y|𝒳,Z,𝒰)-\mu \left\{\theta (Z)+{𝒳}^{T}\beta_{0}\right\}\right]}^{2}\right\}\right|Z=z\right]}{t(z)f_{Z}(z)}.$$

Part (2) of this Theorem gives the asymptotic variance of ${\hat{\theta }}_{\text{AIPW}}(z;\beta )$ when $\hat{\pi }$ is computed under a correctly specified model or $\pi_{i}^{*}=\pi_{i0}$ for all i. The result shows that in such cases, the most efficient AIPW kernel estimator is obtained when E(Y∣𝒳,Z,𝒰) is used for δ(𝒳,Z,𝒰) or when $\delta (𝒳,Z,𝒰;\hat{\eta })$, a model (9) for E(Y∣𝒳,Z,𝒰) is correctly specified.

In contrast to the AIPW approach, inference based on the IPW estimator ${\hat{\theta }}_{IPW}(z)$ is valid only when the selection probabilities are correctly specified, as summarized in the following theorem.

**Theorem 2.** *If model* (3) *for the selection probability is correctly specified and the MAR assumption* (2) *and assumptions I)-IV) above hold, then there exists a sequence of solutions* ${\hat{\theta }}_{IPW}\left(z;\beta_{0}\right)$ *of* (7) *with* β *fixed at* $\beta_{0}$ *such that as* n→∞, h→0, *and* nh→∞,

$$\sqrt{nh}\left\{{\hat{\theta }}_{IPW}\left(z;\beta_{0}\right)-\theta (z)-\frac{1}{2}h^{2}\theta^{\prime \prime }(z)c_{2}(K)+o\left(h^{2}\right)\right\}\to N\left\{0,\Sigma_{\theta ,IPW}(z)\right\}$$

*where* $\Sigma_{\theta ,IPW}(z)$ *is equal to*

$$\frac{c_{0}\left(K^{2}\right)}{t(z)f_{Z}(z)}E\left[\left.r(𝒳,Z)\left\{\frac{var(Y|𝒳,Z,𝒰)}{\pi_{0}(𝒳,Z,𝒰)}+\frac{{\left[E(Y|𝒳,Z,𝒰)-\mu \left\{\theta (Z)+{𝒳}^{T}\beta_{0}\right\}\right]}^{2}}{\pi_{0}(𝒳,Z,𝒰)}\right\}\right|Z=z\right].$$

In the following, we primarily focus on the properties of the AIPW profile estimator ${\hat{\beta }}_{\text{AIPW}}$ in Section 5.1 and compare with those of ${\hat{\beta }}_{IPW}$ in Section 5.2. Lemma 1 and Theorem 3 establish that the AIPW profile estimator of β is consistent and asymptotically normal when either model (9) for the conditional mean of Y given 𝒳, Z, and 𝒰 is correctly specified or model (3) for the missing data mechanism is correctly specified. This property is commonly referred to as double robustness. In contrast, the IPW profile estimator ${\hat{\beta }}_{IPW}$ is inconsistent for β if model (3) for the selection probabilities is misspecified, as shown in Theorem 4. Theorem 3 also establishes that when model (3) for the selection probabilities is correctly specified, then among the class of AIPW profile estimators of β computed under the same working model for $var\left(\epsilon_{\delta }^{*}(\beta ,\theta )\right)$, the one that uses $\delta \left({𝒳}_{i},Z_{i},{𝒰}_{i}\right)=\delta \left({𝒳}_{i},Z_{i},{𝒰}_{i};\hat{\eta }\right)$ with $\hat{\eta }$ computed under a correctly specified model for $E\left(Y_{i}|{𝒳}_{i},Z_{i},{𝒰}_{i}\right)$ has the smallest asymptotic variance. This asymptotic variance aligns with the semiparametric variance bound derived in Section 4 for β in the model defined by restriction (1) on the full data and restriction (2) on the missing data mechanism.

### 5.1. Asymptotic Results of the AIPW Profile Estimator ${\hat{\beta }}_{\text{AIPW}}$

In this subsection, our focus lies on the asymptotic properties of the AIPW profile estimators of β, demonstrating that the optimal estimator among this class has an asymptotic variance equivalent to the semiparametric variance bound derived in Section 4. We define ${\hat{\varphi }}_{\text{AIPW}}(z,\beta )=\frac{\partial {\hat{\theta }}_{\text{AIPW}}(z,\beta )}{\partial \beta }$. Lemma 1 (proved in Supplementary Materials S2) establishes that ${\hat{\varphi }}_{\text{AIPW}}\left(z,\beta_{0}\right)$ converges in probability to $\varphi_{\text{eff}}$ defined in (4) when $V\left[\mu \left\{{𝒳}^{T}\beta +\theta (Z)\right\};\zeta \right]$ is a correctly specified model for $\sigma^{2}(𝒳,Z)=var\left[\epsilon^{*}\left(\beta_{0},\theta \right)|𝒳,Z\right]$, where $\epsilon^{*}\left(\beta_{0},\theta \right)$ is defined in (12). This result, along with the subsequent theorem, is used to argue below that ${\hat{\beta }}_{\text{AIPW}}$ is locally semiparametric efficient.

Let $\varphi_{\text{AIPW}}(z)$ denote the probability limit of ${\hat{\varphi }}_{\text{AIPW}}\left(z,\beta_{0}\right)$ and V denote the probability limit of $V\left[\mu \left\{{𝒳}^{T}\beta +\theta (Z)\right\};\hat{\zeta }\right]$, as n→∞. Let $\epsilon_{\delta }^{*}\left(\beta_{0},\theta \right)$ be $\epsilon_{\delta }^{*}$ evaluated at $\beta_{0}$ and let $\sigma_{\delta }^{2}(𝒳,Z)=var\left[\epsilon_{\delta }^{*}\left(\beta_{0},\theta \right)|𝒳,Z\right]$. Then we have the following Lemma 1.

**Lemma 1.** *Under regularity conditions, we have*

$$\varphi_{\text{AIPW}}\left(z\right)=-\frac{E\left({\left[\mu^{\left(1\right)}\left\{{𝒳}^{T}\beta_{0}+\theta \left(Z\right)\right\}\right]}^{2}V^{-1}𝒳|Z=z\right)}{E\left({\left[\mu^{\left(1\right)}\left\{{𝒳}^{T}\beta_{0}+\theta \left(Z\right)\right\}\right]}^{2}V^{-1}|Z=z\right)}.$$

*In particular, if* $V\left[\mu \left\{{𝒳}^{T}\beta +\theta (Z)\right\};\zeta \right]$ *is a correctly specified model for* $\sigma_{\delta }^{2}(𝒳,Z)$, *then*

$$\varphi_{\text{AIPW}}\left(z\right)=-\frac{E\left({\left[\mu^{\left(1\right)}\left\{{𝒳}^{T}\beta_{0}+\theta \left(Z\right)\right\}\right]}^{2}\sigma_{\delta }^{-2}\left(𝒳,Z\right)𝒳|Z=z\right)}{E\left({\left[\mu^{\left(1\right)}\left\{{𝒳}^{T}\beta_{0}+\theta \left(Z\right)\right\}\right]}^{2}\sigma_{\delta }^{-2}\left(𝒳,Z\right)|Z=z\right)}.$$

Note that $\varphi_{\text{AIPW}}(z)$ is affected by the choice of function δ(𝒳,Z,𝒰) used in the AIPW equations only through the working variance model. If δ(𝒳,Z,𝒰)=E(Y∣𝒳,Z,𝒰) or $\delta (𝒳,Z,𝒰)=\delta (𝒳,Z,𝒰;\hat{\eta })$ and $\hat{\eta }$ is calculated under a correctly specified model (9), a direct result from Lemma 1 is that the limit of the AIPW profile estimating function is proportional to the semiparametric efficient score of β derived in Section 4.

The next theorem establishes the asymptotic distribution of ${\hat{\beta }}_{\text{AIPW}}$. Throughout, we use the subscript δ to emphasize the dependence of ${\hat{\beta }}_{\text{AIPW}}$ and the asymptotic variance of ${\hat{\beta }}_{\text{AIPW}}$ on the choice of function δ(𝒳,Z,𝒰) used in the AIPW equations. Let $\tau^{*}$, $\eta^{*}$, and $\zeta^{*}$ be the probability limits of $\hat{\tau }$, $\hat{\eta }$, and $\hat{\zeta }$. Let $𝒮(R,𝒳,Z,𝒰;\tau )=\partial \text{log}\left[\pi (𝒳,Z,𝒰;\tau {)}^{R}\{1-\pi (𝒳,Z,𝒰;\tau ){\}}^{1-R}\right]/\partial \tau$ be the estimating function for τ and l(Y,𝒳,𝒵,𝒰;η) be the estimating function for η. Denote ${𝒟}^{*}=\mu^{\left(1\right)}\left\{{𝒳}^{T}\beta_{0}+\theta (Z)\right\}V^{-1}\left[\mu \left\{{𝒳}^{T}\beta_{0}+\theta (Z)\right\};\zeta^{*}\right]\tilde{𝒳}$, where $\tilde{𝒳}=𝒳+\varphi_{\text{AIPW}}(Z)$. For any τ, η, β, and θ(⋅), define $\epsilon_{i}(\tau ,\eta ,\beta ,\theta )$ as $\left\{R_{i}\left[Y_{i}-\mu \left\{{𝒳}_{i}^{T}\beta +\theta \left(Z_{i}\right)\right\}\right]\right.-\left.\left\{R_{i}-\pi \left({𝒳}_{i},Z_{i},{𝒰}_{i};\tau \right)\right\}\left[\delta \left({𝒳}_{i},Z_{i},{𝒰}_{i};\eta \right)-\mu \left\{{𝒳}_{i}^{T}\beta +\theta \left(Z_{i}\right)\right\}\right]\right\}/\pi \left({𝒳}_{i},Z_{i},{𝒰}_{i};\tau \right)$. In what follows, for any symmetric matrices A and B, A≥B stands for “A-B is semipositive definite”.

**Theorem 3.** *Suppose that* Equation (6) *uses* ${\hat{\pi }}_{i}$ *computed under a model* (3) *and* $\delta \left({𝒳}_{i},Z_{i},{𝒰}_{i};\hat{\eta }\right)$, where $\hat{\eta }$ *is calculated as in* Section 3. *Under the MAR assumption* (2) *and assumptions I)-IV) above, if either model* (3) *is correct or model* (9) *is correct, then there exists a sequence of solutions* ${\hat{\beta }}_{\text{AIPW},\delta }$ *that satisfy*

(16) $$\sqrt{n}\left\{{\hat{\beta }}_{\text{AIPW},\delta }-\beta_{0}\right\}\to N\left(0,\Omega_{\delta }(V)\right),$$

*where* $\Omega_{\delta }(V)=𝒜(V{)}^{-1}{ℬ}_{\delta }(V)𝒜(V{)}^{-1}$, $𝒜(V)=E\left({\left[\mu^{\left(1\right)}\left\{{𝒳}^{T}\beta_{0}+\theta (Z)\right\}\right]}^{2}V\left[\mu \left\{{𝒳}^{T}\beta_{0}+\right.\right.\right.{\left.\theta (Z)\};\zeta^{*}\right]}^{-1}\tilde{𝒳}{\tilde{𝒳}}^{T})$, *and*

$${ℬ}_{\delta }(V)=var\left\{{𝒟}^{*}\epsilon \left(\tau^{*},\eta^{*},\beta_{0},\theta \right)-E\left[{𝒟}^{*}\frac{\partial }{\partial \tau^{T}}\epsilon \left(\tau^{*},\eta^{*},\beta_{0},\theta \right)\right]E{\left[\frac{\partial }{\partial \tau^{T}}𝒮\left(R,𝒳,Z,𝒰;\tau^{*}\right)\right]}^{-1}\;\left.𝒮\left(R,𝒳,Z,𝒰;\tau^{*}\right)-E\left[{𝒟}^{*}\frac{\partial }{\partial \eta^{T}}\epsilon \left(\tau^{*},\eta^{*},\beta_{0},\theta \right)\right]E{\left[\frac{\partial }{\partial \eta^{T}}l\left(Y,𝒳,𝒵,𝒰;\eta^{*}\right)\right]}^{-1}l\left(Y,𝒳,𝒵,𝒰;\eta^{*}\right)\right\}.\right.$$

Theorem 3 establishes that ${\hat{\beta }}_{\text{AIPW}}$ is consistent and asymptotically normal so long as either model (3) is correct or model (9) is correct but not necessarily both. This is the so-called double-robustness property, which is desirable in practice against model misspecifications.

When model (3) is correctly specified for the selection probability, $E\left[{𝒟}^{*}\partial \epsilon \left(\tau^{*},\eta^{*},\beta_{0},\theta \right)/\partial \tau^{T}\right]=E\left[{𝒟}^{*}\epsilon \left(\tau^{*},\eta^{*},\beta_{0},\theta \right){𝒮}_{\tau }^{T}\right]$, where ${𝒮}_{\tau }$ is the score function of τ, $E\left[\partial 𝒮\left(R,𝒳,Z,𝒰;\tau^{*}\right)/\partial \tau^{T}\right]=E\left[{𝒮}_{\tau }{𝒮}_{\tau }^{T}\right]$, $\pi \left({𝒳}_{i},{𝒵}_{i},{𝒰}_{i};\tau^{*}\right)=\pi_{0}\left({𝒳}_{i},{𝒵}_{i},{𝒰}_{i}\right)$, $E\left[{𝒟}^{*}\partial \epsilon \left(\tau^{*},\eta^{*},\beta_{0},\theta \right)/\partial \eta^{T}\right]=0$, and $\epsilon \left(\tau^{*},\eta^{*},\beta_{0},\theta \right)=\epsilon_{\delta }^{*}\left(\beta_{0},\theta \right)$. Therefore, ${ℬ}_{\delta }(V)$ reduces to $var\left\{{𝒟}^{*}\epsilon_{\delta }^{*}\left(\beta_{0},\theta \right)-E\left[{𝒟}^{*}\epsilon_{\delta }^{*}\left(\beta_{0},\theta \right){𝒮}_{\tau }^{T}\right]E{\left[{𝒮}_{\tau }{𝒮}_{\tau }^{T}\right]}^{-1}{𝒮}_{\tau }\right\}$. Then we have the following two corollaries.

**Corollary 1.** *(i) If* ${\hat{\pi }}_{i}$ *in* (6) *is computed under a correctly specified model* (3), *then* ${ℬ}_{\delta }(V)=var\left\{{𝒟}^{*}\epsilon_{\delta }^{*}\left(\beta_{0},\theta \right)-E\left[{𝒟}^{*}\epsilon_{\delta }^{*}\left(\beta_{0},\theta \right){𝒮}_{\tau }^{T}\right]E{\left[{𝒮}_{\tau }{𝒮}_{\tau }^{T}\right]}^{-1}{𝒮}_{\tau }\right\}$. *(ii) If the true selection probabilities are used in* Equation (6) *instead of* ${\hat{\pi }}_{i}$, *then* (16) *holds with* $\Omega_{\delta }(V)$ *replaced by* ${\tilde{\Omega }}_{\delta }(V)=𝒜(V{)}^{-1}{\tilde{ℬ}}_{\delta }(V)𝒜(V{)}^{-1}$ *and* ${\tilde{ℬ}}_{\delta }(V)=var\left\{{𝒟}^{*}{\tilde{\epsilon }}_{\delta }^{*}\left(\beta_{0},\theta \right)\right\}$.

Part (ii) of Corollary 1 holds since $E\left[{𝒟}^{*}\partial \epsilon \left(\tau^{*},\eta^{*},\beta_{0},\theta \right)/\partial \eta^{T}\right]=0$ and ${𝒮}_{\tau }=0$ when the true selection probabilities are used in (6). Notice that in (i), ${𝒟}^{*}\epsilon_{\delta }^{*}\left(\beta_{0},\theta \right)-E\left[{𝒟}^{*}\epsilon_{\delta }^{*}\left(\beta_{0},\theta \right){𝒮}_{\tau }^{T}\right]E{\left[{𝒮}_{\tau }{𝒮}_{\tau }^{T}\right]}^{-1}{𝒮}_{\tau }$ is the residual from the population least squares of ${𝒟}^{*}\epsilon_{\delta }^{*}\left(\beta_{0},\theta \right)$ on ${𝒮}_{\tau }$, and residual variances are always less than or equal to the variance of the outcomes in a regression; therefore, ${ℬ}_{\delta }(V)\leq {\tilde{ℬ}}_{\delta }(V)$ whenever model (3) is correct. Thus, Corollary 1 implies that using $\sqrt{n}$–consistent estimates of the selection probabilities even when these are known yields efficiency gains for estimating β. This property has also been observed for parametric regression estimation by Robins et al. (1994) [29]. The asymptotic variance of ${\hat{\beta }}_{\text{AIPW},\delta }$ varies with different choices of δ and working model V. The following corollary implies that for a fixed working model V, ${\hat{\beta }}_{\text{AIPW},\delta }$ has the smallest variance when $\delta_{\text{opt}}(𝒳,Z,𝒰)=E(Y|𝒳,Z,𝒰)$ is used in the augmentation term. Furthermore, if we correctly specify the working model V as $\sigma_{\delta_{\text{opt}}}^{2}$, the asymptotic variance of ${\hat{\beta }}_{\text{AIPW},\delta }$ is minimized and the semiparametric variance bound derived in Section 4 is achieved.

**Corollary 2.** *If* Equation (6) *uses* ${\hat{\pi }}_{i}$ *computed under a correctly specified model* (3) *or the true selection probabilities, we have the following: (i)* $\Omega_{\delta_{\text{opt}}}(V)={\tilde{\Omega }}_{\delta_{\text{opt}}}(V)$ *when* $\delta_{\text{opt}}(𝒳,Z,𝒰)=E(Y|𝒳,Z,𝒰)$. *(ii) For any* δ, $\Omega_{\delta_{\text{opt}}}(V)\leq \Omega_{\delta }(V)$ *and* $\Omega_{\delta_{\text{opt}}}(V)\leq {\tilde{\Omega }}_{\delta }(V)$. *Furthermore*, ${ℬ}_{\delta_{\text{opt}}}(V)={\tilde{ℬ}}_{\delta_{\text{opt}}}(V)=var\left\{{𝒟}^{*}\epsilon_{\delta_{\text{opt}}}^{*}\left(\beta_{0},\theta \right)\right\}$. *(iii) For any* V, $\Omega_{\delta_{\text{opt}}}(V)\geq \Omega_{\delta_{\text{opt}}}\left(\sigma_{\delta_{\text{opt}}}^{2}\right)$. *Furthermore*, $\Omega_{\delta_{\text{opt}}}\left(\sigma_{\delta_{\text{opt}}}^{2}\right)=𝒜{\left(\sigma_{\delta_{\text{opt}}}^{2}\right)}^{-1}$.

Part (i) of Corollary 2 shows that when E(Y∣𝒳,Z,𝒰) is employed as the δ function, then estimating the selection probabilities does not yield efficiency gains for estimating β compared to using the true selection probabilities when they are known. As indicated by part (ii) of Corollary 2, among all δ functions, $\delta_{\text{opt}}(𝒳,Z,𝒰)=E(Y|𝒳,Z,𝒰)$ is the optimal one to achieve the smallest asymptotic variance of $\overset{ˆ}{\beta }$. In part (iii), because $\sigma_{\delta_{opt}}^{2}=var\left[\epsilon^{*}\left(\beta_{0},\theta \right)|𝒳,Z\right]$ and by Lemma 1 $\tilde{𝒳}=𝒳-\varphi_{\text{eff}}(Z)$ when $V=\sigma_{\delta_{\text{opt}}}^{2}$, it follows that $\Omega_{\delta_{\text{opt}}}\left(\sigma_{\delta_{\text{opt}}}^{2}\right)$ is equal to the semiparametric variance bound (13) for RAL estimators of β. We therefore conclude from Theorem 3 and Corollary 2 that the profile AIPW estimator ${\hat{\beta }}_{\text{AIPW}}$ is locally semiparametric efficient in the semiparametric model defined by restriction (1) on the full data and restrictions (2) and (3) on the selection probabilities, at the models (4) and (9). That is, it is consistent and asymptotically normal if (1), (2), and (3) hold regardless of whether (4) and (9) hold, and it has asymptotic variance equal to the semiparametric variance bound if, in addition, models (4) and (9) hold.

Corollary 1 and Corollary 2 present the properties of ${\hat{\beta }}_{\text{AIPW}}$ when model (3) is correctly specified for the selection probability π(𝒳,Z,𝒰) or the true selection probabilities are used. Otherwise, if one can achieve a valid specification for model (9), ${\hat{\beta }}_{\text{AIPW}}$ is still consistent and asymptotically normal due to its double-robustness property, established in Theorem 3. In this situation, $E\left[{𝒟}^{*}\partial \epsilon \left(\tau^{*},\eta^{*},\beta_{0},\theta \right)/\partial \tau^{T}\right]=0$, and that leads to the following Corollary 3.

**Corollary 3.** *If* Equation (6) *uses* $\delta \left({𝒳}_{i},Z_{i},{𝒰}_{i};\hat{\eta }\right)$ *under a correctly specified model* (9), *then* ${ℬ}_{\delta }(V)$ *simplifies to*

$${ℬ}_{\delta }(V)=var\left\{{𝒟}^{*}\epsilon \left(\tau^{*},\eta^{*},\beta_{0},\theta \right)-E\left[{𝒟}^{*}\frac{\partial }{\partial \eta^{T}}\tilde{\epsilon }\left(\tau^{*},\eta^{*},\beta_{0},\theta \right)\right]\;\left.E{\left[\frac{\partial }{\partial \eta^{T}}l\left(Y,𝒳,𝒵,𝒰;\eta^{*}\right)\right]}^{-1}l\left(Y,𝒳,𝒵,𝒰;\eta^{*}\right)\right\}\right.$$

Following Theorem 3, we can estimate the variance of AIPW profile estimator ${\hat{\beta }}_{\text{AIPW}}$ using a sandwich formula after invoking a uniform law of large numbers. Specifically, let $\hat{\delta }(𝒳,Z,𝒰)=\delta (𝒳,Z,𝒰;\hat{\eta })$, ${\hat{𝒳}}_{i}={𝒳}_{i}+{\hat{\varphi }}_{\text{AIPW}}\left(Z_{i},{\hat{\beta }}_{\text{AIPW},\hat{\delta }}\right)$, where ${\hat{\varphi }}_{\text{AIPW}}(\cdot ,\cdot )$ is as defined in the first paragraph of Section 5.2, ${\hat{V}}_{i}=V\left[\mu \left\{{𝒳}_{i}^{T}{\hat{\beta }}_{\text{AIPW},\hat{\delta }}+{\hat{\theta }}_{\text{AIPW},\hat{\delta }}\left(Z_{i};{\hat{\beta }}_{\text{AIPW},\hat{\delta }}\right)\right\};\hat{\zeta }\right]$, and ${\hat{\mu }}_{i}^{\left(1\right)}=\mu^{\left(1\right)}\left\{{𝒳}_{i}^{T}{\hat{\beta }}_{\text{AIPW},\hat{\delta }}+{\hat{\theta }}_{\text{AIPW},\hat{\delta }}\left(Z_{i};{\hat{\beta }}_{\text{AIPW},\hat{\delta }}\right)\right\}$. $\Omega_{\delta }(V)$ can be estimated consistently when either model (3) is correct or model (9) is correct, as ${𝒜}_{n}(\hat{V}{)}^{-1}{\tilde{ℬ}}_{\hat{\delta },n}(\hat{V}){𝒜}_{n}(\hat{V}{)}^{-1}$ where

$${𝒜}_{n}\left(\hat{V}\right)=\frac{1}{n}\sum_{i=1}^{n}{\left\{{\hat{\mu }}_{i}^{\left(1\right)}\right\}}^{2}{\hat{V}}_{i}^{-1}{\hat{𝒳}}_{i}{\hat{𝒳}}_{i}^{T},$$

and

$${\tilde{ℬ}}_{\hat{\delta },n}(\hat{V})=\frac{1}{n}\sum_{i=1}^{n}\left\{{\hat{𝒟}}_{i}\epsilon_{i}\left\{\hat{\tau },\hat{\eta },{\hat{\beta }}_{\text{AIPW},{\hat{\delta }}^{\prime }}{\hat{\theta }}_{\text{AIPW},\hat{\delta }}\left(\cdot ;{\hat{\beta }}_{\text{AIPW},\hat{\delta }}\right)\right\}\right.\;-\hat{E}\left[{𝒟}^{*}\frac{\partial }{\partial \tau^{T}}\epsilon \left(\tau^{*},\eta^{*},\beta_{0},\theta \right)\right]\hat{E}{\left[\frac{\partial }{\partial \tau^{T}}𝒮\left(R,𝒳,Z,𝒰;\tau^{*}\right)\right]}^{-1}𝒮\left(R_{i},{𝒳}_{i},Z_{i},{𝒰}_{i};\hat{\tau }\right)\;{\left.-\hat{E}\left[{𝒟}^{*}\frac{\partial }{\partial \eta^{T}}\epsilon \left(\tau^{*},\eta^{*},\beta_{0},\theta \right)\right]\hat{E}{\left[\frac{\partial }{\partial \eta^{T}}l\left(Y,𝒳,𝒵,𝒰;\eta^{*}\right)\right]}^{-1}l\left(Y_{i},{𝒳}_{i},{𝒵}_{i},{𝒰}_{i};\hat{\eta }\right)\right\}}^{⊗2}$$

with

$${\hat{𝒟}}_{i}={\hat{\mu }}_{i}^{\left(1\right)}{\hat{V}}_{i}^{-1}\left\{{𝒳}_{i}+\frac{\partial \hat{\theta }\left(Z_{i},{\hat{\beta }}_{\text{AIPW},\hat{\delta }}\right)}{\partial \beta^{T}}\right\},\;\hat{E}\left[{𝒟}^{*}\frac{\partial }{\partial \tau^{T}}\epsilon \left(\tau^{*},\eta^{*},\beta_{0},\theta \right)\right]=\frac{1}{n}\sum_{i=1}^{n}{\hat{𝒟}}_{i}\frac{\partial }{\partial \tau^{T}}\epsilon_{i}\left\{\hat{\tau },\hat{\eta },{\hat{\beta }}_{\text{AIPW},\hat{\delta }},{\hat{\theta }}_{\text{AIPW},\hat{\delta }}\left(\cdot ;{\hat{\beta }}_{\text{AIPW},\hat{\delta }}\right)\right\},\;\hat{E}\left[\frac{\partial }{\partial \tau^{T}}𝒮\left(R,𝒳,Z,𝒰;\tau^{*}\right)\right]=\frac{1}{n}\sum_{i=1}^{n}\frac{\partial }{\partial \tau^{T}}𝒮\left(Y_{i},{𝒳}_{i},{𝒵}_{i},{𝒰}_{i};\hat{\tau }\right)\;\hat{E}\left[{𝒟}^{*}\frac{\partial }{\partial \eta^{T}}\epsilon \left(\tau^{*},\eta^{*},\beta_{0},\theta \right)\right]=\frac{1}{n}\sum_{i=1}^{n}{\hat{𝒟}}_{i}\frac{\partial }{\partial \eta^{T}}\epsilon_{i}\left\{\hat{\tau },\hat{\eta },{\hat{\beta }}_{\text{AIPW},\hat{\delta }},{\hat{\theta }}_{\text{AIPW},\hat{\delta }}\left(\cdot ;{\hat{\beta }}_{\text{AIPW},\hat{\delta }}\right)\right\},\;\hat{E}\left[\frac{\partial }{\partial \eta^{T}}l\left(Y,𝒳,𝒵,𝒰;\eta^{*}\right)\right]=\frac{1}{n}\sum_{i=1}^{n}\frac{\partial }{\partial \eta^{T}}l\left(Y_{i},{𝒳}_{i},{𝒵}_{i},{𝒰}_{i};\hat{\eta }\right).$$

### 5.2. Asymptotic Results of the IPW Profile Estimator ${\hat{\beta }}_{IPW}$

In comparison, we present the asymptotic properties of the IPW profile estimator ${\hat{\beta }}_{IPW}$ in this subsection. The results stated in the next lemma and theorem imply that ${\hat{\beta }}_{IPW}$ is generally not efficient. This is not surprising after noticing that ${\hat{\beta }}_{IPW}$ actually solves the AIPW equation that uses a fixed function $\delta (𝒳,Z,𝒰)=\mu \left\{{𝒳}^{T}\beta_{0}+\theta (Z)\right\}$; however, this specific δ function is not the optimal one among the AIPW class and also makes the IPW profile estimator ${\hat{\beta }}_{IPW}$ lose the double-robustness property. In order for ${\hat{\beta }}_{IPW}$ to be consistent and asymptotically normal, model (3) for the selection probability π(𝒳,Z,𝒰) needs to be correctly specified.

**Lemma 2.** *Let* ${\hat{\varphi }}_{IPW}(z,\beta )=\partial {\hat{\theta }}_{IPW}(z,\beta )/\partial \beta$ *be the partial derivative of the final IPW kernel estimator of* θ *with respect to* β, *and let* $\varphi_{IPW}(z)$ *be the probability limit of* ${\hat{\varphi }}_{IPW}(z,\beta )$ *as* n→∞. *Then*

$$\varphi_{IPW}\left(z\right)=-\frac{E\left({\left[\mu^{\left(1\right)}\left\{{𝒳}^{T}\beta_{0}+\theta \left(Z\right)\right\}\right]}^{2}V^{-1}𝒳|Z=z\right)}{E\left({\left[\mu^{\left(1\right)}\left\{{𝒳}^{T}\beta_{0}+\theta \left(Z\right)\right\}\right]}^{2}V^{-1}|Z=z\right)}.$$

*If* $V\left[\mu \left\{{𝒳}^{T}\beta +\theta (Z)\right\};\zeta \right]$ *is correctly specified for* $\sigma_{IPW}^{2}=var\left[\epsilon_{IPW}^{*}\left(\beta_{0},\theta \right)|𝒳,Z\right]$, *where* $\epsilon_{\text{IPW}}^{*}\left(\beta_{0},\theta \right)=R\cdot {\left\{\pi_{0}(𝒳,Z,𝒰)\right\}}^{-1}\epsilon \left(\beta_{0},\theta \right)$, *then*

$$\varphi_{IPW}(z)=-\frac{E\left({\left[\mu^{\left(1\right)}\left\{{𝒳}^{T}\beta_{0}+\theta (Z)\right\}\right]}^{2}𝒳/\sigma_{IPW}^{2}|Z=z\right)}{E\left({\left[\mu^{\left(1\right)}\left\{{𝒳}^{T}\beta_{0}+\theta (Z)\right\}\right]}^{2}/\sigma_{IPW}^{2}|Z=z\right)}.$$

Theorem 4 on the asymptotic distribution of ${\hat{\beta }}_{IPW}$ follows directly from Lemma 2, Theorem 3, and Corollaries 1 and 2 with $\delta (𝒳,Z,𝒰)=\mu \left\{{𝒳}^{T}\beta_{0}+\theta (Z)\right\}$.

**Theorem 4.** *Under the assumptions of Theorem 3, if model* (3) *holds, we have*

$$\sqrt{n}\left({\hat{\beta }}_{IPW}-\beta_{0}\right)\to N\left\{0,\Omega_{IPW}(V)\right\},$$

*where*

$$\Omega_{IPW}(V)=𝒜(V{)}^{-1}{ℬ}_{IPW}\left(V\right){\left\{𝒜(V{)}^{-1}\right\}}^{T},\;{ℬ}_{IPW}(V)=var\left\{{𝒟}^{*}\epsilon_{IPW}^{*}\left(\beta_{0},\theta \right)-E\left[{𝒟}^{*}\epsilon_{IPW}^{*}\left(\beta_{0},\theta \right){𝒮}_{\tau }^{T}\right]E{\left[{𝒮}_{\tau }{𝒮}_{\tau }^{T}\right]}^{-1}{𝒮}_{\tau }\right\}.$$

In general, ${𝒟}^{*}\epsilon_{IPW}^{*}\left(\beta_{0},\theta \right)-E\left[{𝒟}^{*}\epsilon_{IPW}^{*}\left(\beta_{0},\theta \right){𝒮}_{\tau }^{T}\right]E{\left[{𝒮}_{\tau }{𝒮}_{\tau }^{T}\right]}^{-1}{𝒮}_{\tau }$ is not proportional to the efficient score derived in Section 4, and thus, the IPW profile estimator ${\hat{\beta }}_{IPW}$ is generally an inefficient estimator.

## 6. Simulations

In this section, we conduct simulation studies to compare the finite-sample performance of the AIPW and IPW kernel–profile estimators for θ(⋅) and β, as well as the naive approach which solves unweighted kernel–profile estimating equations based on units with no missing data (complete cases). We generate data according to the spirit of a two-stage study design, where in the first stage we observe the covariates of interest X (e.g., treatment), nuisance covariates Z (e.g., age), and auxiliary variables U for every subject, while in the second stage we only measure the outcome of interest Y on a subset resampled from the first-stage cohort according to some selection probabilities, which depend on the first-stage variables, especially U. For each replication, we generate random samples of (X,Z,U,Y,R), where Z is generated from a Uniform(0, 1) distribution, X is generated from $\text{Norm}\left\{(Z-0.5{)}^{2},1\right\}$, U is generated from a $\text{Uniform}(0,6)+\text{Norm}\left(X,0.05^{2}\right)+\text{Norm}\left(Z,0.05^{2}\right)$, and the outcome Y is generated from a normal distribution with mean

(17) $$E(Y|X,Z,U)=X\beta_{1}+m(Z)+U\beta_{2}$$

and variance $\sigma_{Y|X,Z,U^{\prime }}^{2}$, where $\beta_{1}=\beta_{2}=1,\sigma_{Y|X,Z,U}^{2}=1$, $m(Z)=2\cdot F_{8,8}(Z)$, and $F_{p,q}(Z)=\Gamma (p+q)\{\Gamma (p)\Gamma (q){\}}^{-1}Z^{p-1}(1-Z{)}^{q-1}$, a unimodal function. Note that Z is correlated with X, U, and Y, while U is correlated with X, Z, and Y. We generate R, the selection indicator, according to

(18) $$\text{logit}\left\{\pi_{i}\right\}=\tau_{0}+\tau_{1}\cdot \left(U_{i}-a_{1}\right)I\left(a_{1}<U_{i}\leq a_{2}\right)+\tau_{1}\cdot \left(a_{2}-a_{1}\right)I\left(U_{i}>a_{2}\right)$$

where $\pi_{i}=P\left(R_{i}=1|X_{i},Z_{i},U_{i}\right)$ is the probability that subject i is selected to the second stage, $\tau_{0}=-2$, $\tau_{1}=1$, $a_{1}=0.5$, and $a_{2}=5.5$. Based on this selection mechanism, the Monte Carlo median missing percentage of the outcome Y is around 35%. Since the selection probability depends on U only, the assumption of missing at random holds. Note that

E(Y∣X,Z)=Xβ+θ(Z),

where the true β=2, and true θ(Z)=m(Z)+Z+3. Our primary interest lies in estimating β and the nonparametric curve θ(z).

We generated 100 replications with sample sizes n=500 or n=1000 in each dataset. For each simulated dataset, we applied the naive approach, which uses the complete cases directly, as well as the IPW and the proposed AIPW kernel–profile methods to estimate the nonparametric function θ(⋅) and the semiparametric parameter β. We employed the generalized EBBS method to select the optimal local bandwidth.

**Figure 1.**
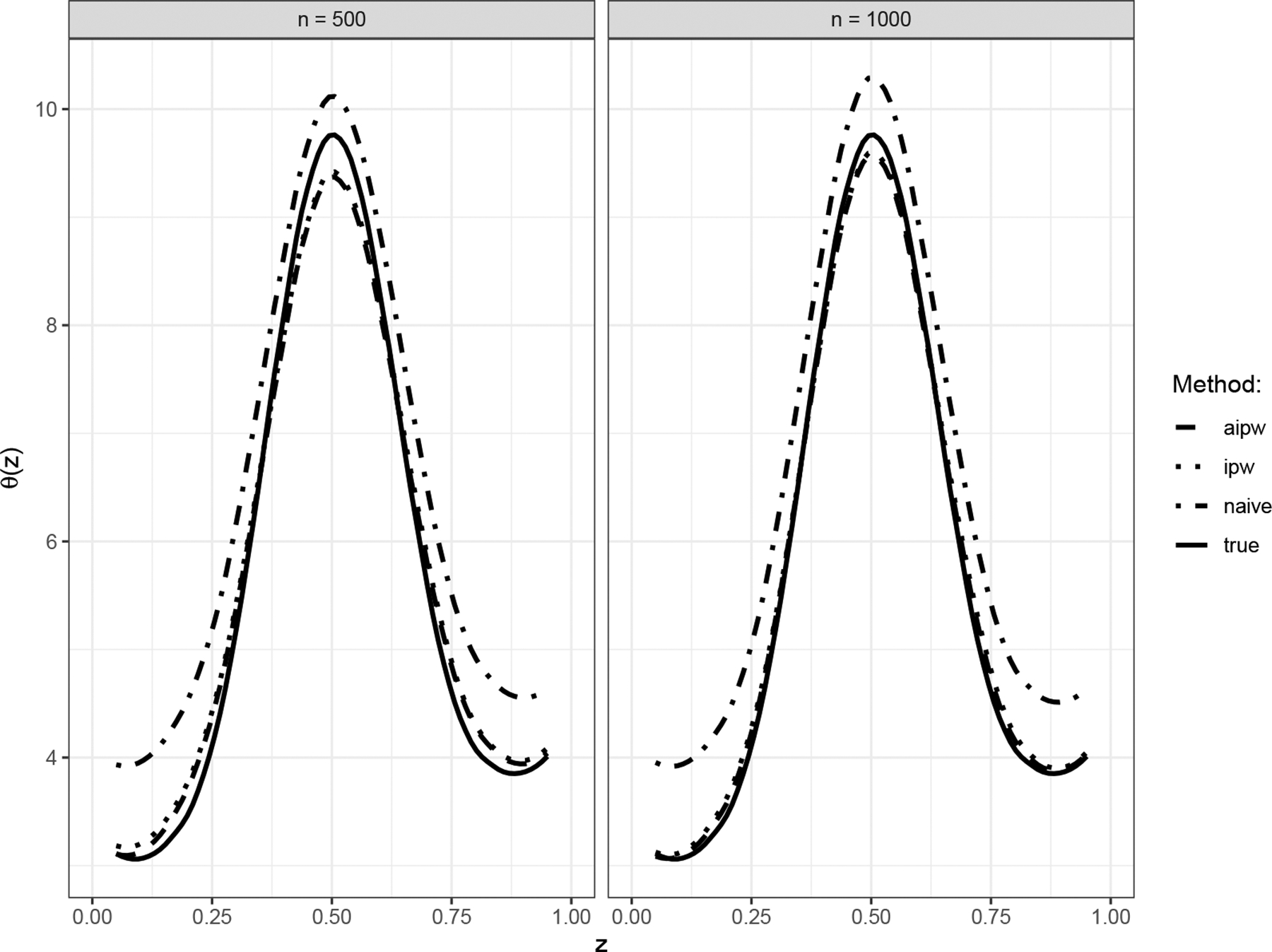
The true θ(z) and the estimated nonparametric functions $\hat{\theta }(z)$ through naive, IPW, and AIPW kernel estimating equations based on 100 replications.

Figure 1 displays the average estimated nonparametric functions $\hat{\theta }(\cdot )$ over 100 replications using the naive, IPW, and AIPW approaches. The plot shows the estimates when the weighted kernel–profile estimating equations use the true $\pi_{i0}$ and $E\left[Y|X=x_{i},Z=z_{i},U=u_{i}\right]$. The IPW and AIPW kernel estimates closely matched the true curve θ(⋅), while the naive approach produced an estimate biased away from the true curve.

**Figure 2.**
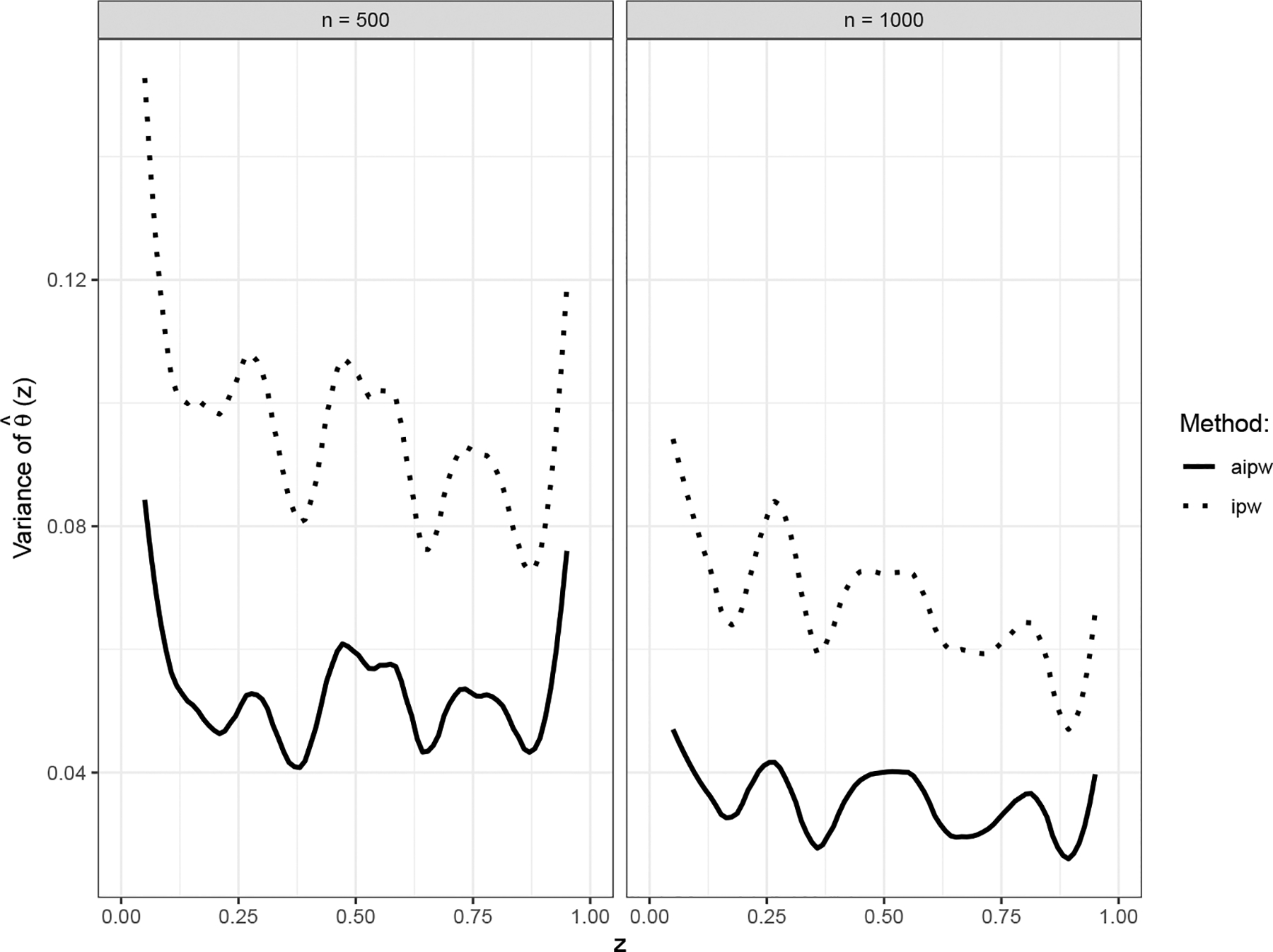
Empirical pointwise variance of the IPW and AIPW estimated nonparametric functions $\hat{\theta }(z)$ based on 100 replications.

Table 1 summarizes the performance of each kernel estimator using integrated relative bias, integrated empirical standard error (SE), integrated estimated SE, and empirical mean integrated squared error (MISE), over the support of Z. The integrated estimated SEs are close to the integrated empirical SEs. As expected, the naive kernel estimate exhibits a much larger relative bias than the IPW and AIPW kernel estimates. The AIPW kernel estimate using the true $\pi_{i0}$ and $E\left[Y|X=x_{i},Z=z_{i},U=u_{i}\right]$ is optimal, having both a smaller SE and a smaller MISE compared to the IPW kernel estimate. The efficiency gain for $\hat{\theta }(z)$ in terms of MISE is about 40%. Figure 2 illustrates the estimated pointwise variance of ${\hat{\theta }}_{IPW}(\cdot )$ and ${\hat{\theta }}_{\text{AIPW}}(\cdot )$ based on 100 replications. It shows that the AIPW approach is more efficient than the IPW approach at each point z in this simulation setup.

In Table 1, we also evaluate the performance of each profile estimator using the averaged relative bias, empirical SE, estimated SE, and mean squared error (MSE). For all estimates, the estimated SEs are close to the empirical SEs, demonstrating that the sandwich estimator we proposed for the variance of $\hat{\beta }$ in Section 5.2 performs well. The bias of ${\hat{\beta }}_{\text{naive}}$ was relatively large. When the true $\pi_{i0}$ or consistent estimates of $\pi_{i0}$ was used, ${\hat{\beta }}_{IPW}$ had very little bias. Otherwise, ${\hat{\beta }}_{IPW}$ was biased. By contrast, the simulation results in Table 1 demonstrate the double robustness of the AIPW kernel-profile estimators. We computed ${\hat{\theta }}_{\text{AIPW}}(\cdot )$ and ${\hat{\beta }}_{\text{AIPW}}$ under three scenarios: (i) with an incorrectly specified model of $\pi_{i0}$, e.g., $\tau_{0}^{\prime }+\tau_{1}^{\prime }\cdot \left|X_{i}\right|+\tau_{2}^{\prime }\cdot Z_{i}$ on the right side of (18), but with $\delta_{i0}$ computed from a correctly specified model (17); (ii) with $\delta_{i0}$ computed from an incorrectly specified model, e.g., $\beta_{0}^{\prime }+\beta_{1}^{\prime }Z+\beta_{2}^{\prime }X$ in the right side of (17), but with $\pi_{i0}$ derived from the correctly specified model (18); and (iii) with both ${\hat{\pi }}_{i}$ and ${\overset{ˆ}{\delta }}_{i}$ computed from incorrectly specified models respectively. When either the true $\pi_{i0}$ or the true $E\left[Y|X=x_{i},Z=z_{i},U=u_{i}\right]$ was used or the consistent estimates were used, as in scenarios (i) and (ii), the AIPW kernel-profile estimates were still close to the true values. However, when both were incorrectly specified, as in scenario (iii), the AIPW estimates were subject to biases. Comparing the IPW estimator using the true $\pi_{i0}$ and the AIPW estimator using both true $\pi_{i0}$ and true $E\left[Y|X=x_{i},Z=z_{i},U=u_{i}\right]$, the SE and the MSE of the IPW profile estimate are much larger than the AIPW profile estimate. Under the true $\pi_{i0}$ model and the true $E\left[Y|X=x_{i},Z=z_{i},U=u_{i}\right]$ model, the efficiency gain of ${\hat{\beta }}_{\text{AIPW}}$ in terms of the MSE is about 47% relative to ${\hat{\beta }}_{IPW}$.

## 7. Application to the SPECT Data

To illustrate the proposed methods, we applied the AIPW kernel–profile estimating equations to analyze the SPECT data described in Section 1. Our primary objective was to investigate the potential risk factors of myocardial ischemia while controlling for patient age. The data analysis indicated that the risk of myocardial ischemia varies nonlinearly with age, prompting us to model the age effect nonparametrically. The data were collected at the Radiology Clinic of the Nuclear Imaging Group at Cedars Sinai Medical Center. Since myocardial ischemia is relatively rare in younger individuals, we focused on 6185 patients aged 45 and older. This two-stage study involved all patients undergoing EBCT in the first stage. Based on the initial results and other health variables, 458 patients with high-risk factors for coronary artery disease were referred by their doctors to undergo SPECT in the second stage.

The SPECT test serves as the gold standard for screening myocardial ischemia. Consequently, we assumed that the 5727 (93%) patients who did not undergo SPECT were unaware of their true myocardial ischemia status, resulting in missing outcomes. We employed model (1) with myocardial ischemia status as the outcome variable. The covariates of interest include patient age, a continuous variable, and patient gender, smoking status, presence of chest pain, high blood pressure status, and cholesterol status, all binary variables. While polynomial regression with terms like *age*^2^ or *age*^3^ can model nonlinear relationships, it may overlook local variations. Kernel-profile estimating equations offer greater flexibility, capturing nuances in the data that fixed polynomial terms might miss. We use a partially linear logistic regression model to estimate the probability of myocardial ischemia, with patient gender, smoking status, presence of chest pain, high-blood-pressure status, and cholesterol status serving as linear predictors, while the effect of age was modeled nonparametrically. The bandwidth was determined using the generalized EBBS method.

**Figure 3.**
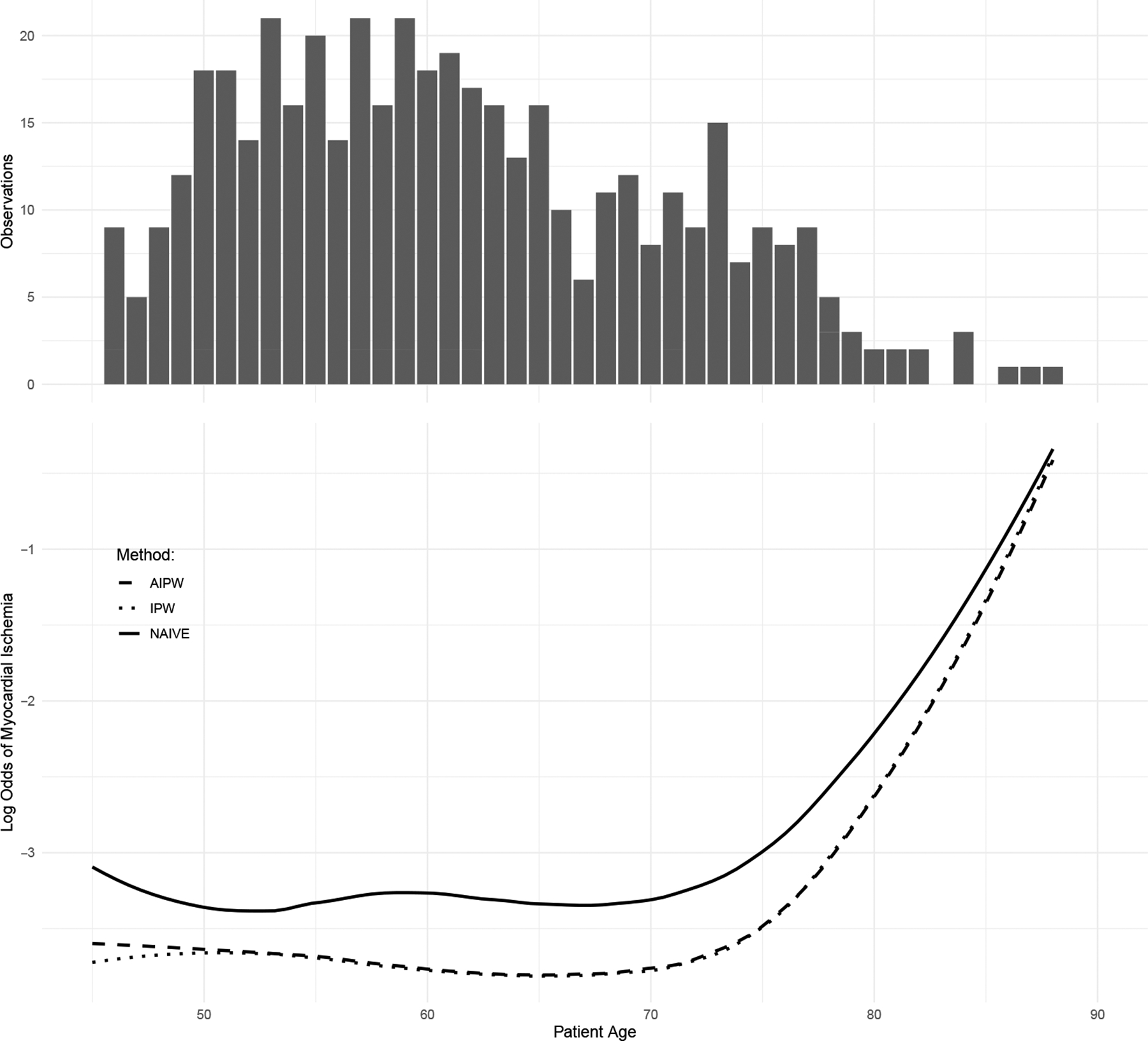
Estimate of θ (age) for the risk of myocardial ischemia controlled for other potential risk factors and confounders.

Figure 3 presents the estimated nonparametric curve of the risk of myocardial ischemia in relation to patient age. The curves estimated by the IPW and AIPW methods closely resemble each other, whereas the naive unweighted approach tends to overestimate the risk of myocardial ischemia. Given that primarily high-risk patients underwent the SPECT exam, relying solely on complete cases using the naive approach is likely to lead to bias in estimating the nonparametric relationship between myocardial ischemia risk and age, as well as the relationship on the logistic scale with other covariates. Our analysis utilizing IPW and AIPW kernel–profile estimating equations suggests that the risk of myocardial ischemia nonlinearly increases with age, with a notable change point around age 70.

Table 2 displays the estimates of regression coefficients from the model, along with the corresponding p-values. Once more, the IPW and AIPW profile-kernel estimates exhibit similarity and contrast with the naive estimates. Our weighted analysis indicates that women tend to have a lower risk of myocardial ischemia compared to men, while patients who smoke or experience chest pain, high blood pressure, or high cholesterol tend to have a heightened risk of myocardial ischemia. Particularly noteworthy is the statistically significant impact of gender and high blood pressure on myocardial ischemia risk. According to the AIPW analysis, patients experiencing high blood pressure have odds approximately 3.6 times higher of developing myocardial ischemia. Additionally, men have approximately 5 times higher odds (p=0.044) of experiencing myocardial ischemia compared to women.

## 8. Discussion

In this paper, we propose weighted local polynomial kernel-profile estimation methods for generalized semiparametric partially linear regression in cases where outcomes are missing at random while auxiliary variables exist. We demonstrate that the estimators based on the IPW and AIPW kernel-profile estimating equations are consistent and asymptotically normal if the selection probability model π is correctly specified. When the π model is misspecified, the IPW approach fails to provide consistent estimators. However, the AIPW kernel-profile estimators maintain consistency and asymptotic normality if either the π model or the model for E(Y∣𝒳,Z,𝒰) is correctly specified. This double-robustness property of the AIPW approach allows investigators two avenues for making valid inferences. Furthermore, the AIPW kernel-profile estimators optimally utilize information in observed data: when both the $\pi_{i}$ selection probability model and the E(Y∣𝒳,Z,𝒰) model are correctly specified, the corresponding AIPW kernel-profile estimators are the most efficient among its class, with the AIPW profile estimator achieving the semiparametric efficiency bound. User-friendly R code has been uploaded to GitHub, which can be accessed at https://github.com/Team-Wang-Lab/AIPWKPEE.git (accessed on 23 July 2024).

When E(Y∣𝒳,Z,𝒰) is not correctly specified, Wang et al. (2010) [20] proposed a modified AIPW kernel estimator for nonparametric regression, which is guaranteed to be more efficient than the IPW kernel estimator and meanwhile also doubly robust. The same idea can be applied to the semiparametric IPW and AIPW kernel-profile estimators proposed in this paper. The IPW and AIPW kernel-profile estimating equations provide consistent estimators when the selection probability model π is correctly specified and is bounded away from 0. However, when some $\pi^{\prime }$s are close to 0 with moderate sample sizes, the associated large weights can dramatically inflate a few observations. Therefore, the IPW and AIPW estimators might not perform well and cause unstable results. Special caution is hence needed when applying the proposed methods to studies when the selection probability is very small for some sample units.

The proposed method can be extended to the situation where multiple covariates need to be modeled nonparametrically, e.g., using additive models. For simplicity, we concentrate on local linear kernel estimators for the nonparametric function, but these methods can be readily extended to higher-order local polynomial kernel regression with similar asymptotic results. Although we adopt a parametric model for the missingness probability $\pi_{i}$ in this paper, future research could explore the nonparametric estimation of $\pi_{i}$ and its impact on the semiparametric efficiency of both IPW and AIPW profile estimators of β. However, fully nonparametric modeling of $\pi_{i}$ is challenged by the curse of dimensionality, particularly when $\pi_{i}$ depends on a set of covariates.

If some covariates of interest among 𝒳 and Z are missing in addition to Y, the general AIPW profile-kernel theory remains applicable, but the efficient score and efficiency bound may change, and adjustments may be necessary for the corresponding estimating procedure. This topic exceeds the scope of our current paper, and further research is needed on complex scenarios where missingness occurs in both outcomes and covariates. We assume in this paper that the outcome is missing at random (MAR). However, justification for the MAR assumption may be required in observational studies, particularly when the missing data mechanism is not well understood. The literature is substantial on statistical methods for parametric regression in the presence of not missing at random (NMAR) for specific cases. Extending these methods and our proposed methods to fit the semiparametric model (1) under NMAR conditions represents a future research direction.

There are several challenges in the practical implementation. For example, bandwidth selection can be time-consuming in a grid search without any prior knowledge. To reduce the computational burden, one can initially search for the bandwidth on a coarser grid, followed by a finer grid search. Additionally, selecting the appropriate auxiliary variable(s) and specifying the π model are crucial factors. Insights from experts would also be beneficial.

## Supplementary Material

supplementary materials

Supplementary Materials: The following supporting information can be downloaded at https://www.mdpi.com/article/10.3390/stats7030056/s1. These include detailed regularity conditions; Section S1: derivation of the semiparametric efficient score presented in Section 4; Section S2: proof of Lemmas 1 and 2; Section S3: proof of Theorem 3 and Corollary 2; and Section S4: additional simulation results and a sensitivity analysis for the application.

## Figures and Tables

**Table 1.** Simulation results of the naive, IPW, and AIPW kernel–profile estimates based on 100 replications (sample size n=500).

	Kernel Estimator of θ(⋅)				Profile Estimator of β			
	Relative Bias^1^	EMP S.E.^2^	EST S.E.^3^	EMP MISE^4^	Bias of $\hat{\beta }$	EMP S.E.	EST S.E.	EMP MSE
**Naive Estimator**	0.166	0.231	0.226	0.672	0.120	0.108	0.102	0.070
**IPW Estimator**								
True π	0.066	0.338	0.308	0.167	0.055	0.140	0.124	0.019
Consistent $\hat{\pi }$	0.064	0.329	0.311	0.158	0.051	0.130	0.125	0.017
Wrong π	0.162	0.222	0.226	0.638	0.137	0.115	0.102	0.088
**AIPW Estimator**								
True π and δ^5^	0.049	0.228	0.228	0.100	0.041	0.099	0.101	0.010
Consistent $\hat{\pi }$ and consistent $\hat{\delta }$	0.047	0.231	0.233	0.092	0.040	0.099	0.100	0.010
Wrong π and consistent $\hat{\delta }$	0.048	0.124	0.213	0.096	0.046	0.110	0.092	0.012
Consistent $\hat{\pi }$ and wrong δ	0.077	0.399	0.404	0.218	0.067	0.169	0.153	0.029
Both wrong	0.162	0.279	0.286	0.653	0.109	0.125	0.114	0.060

Note:

1 relative bias is defined as $\int |\hat{\text{bias}}\{\hat{\theta }(z)\}/\theta (z)|dF(z)$;

2 EMP S.E. is the empirical S.E., defined as $\int {\hat{SE}}_{EMP}\{\hat{\theta }(z)\}dF(z)$, where ${\hat{SE}}_{EMP}\{\hat{\theta }(z)\}$ is the sampling S.E. of the replicated $\hat{\theta }(z);$

3 EST S.E. is the estimated S.E., defined as $\int {\hat{SE}}_{EST}\{\hat{\theta }(z)\}dF(z)$, where ${\hat{SE}}_{EST}\{\hat{\theta }(z)\}$ is the sampling average of the replicated sandwich estimates $\hat{SE}\{\hat{\theta }(z)\};$

4 EMP MISE is the empirical MISE, defined as $\int \{\hat{\theta }(z)-\theta (z){\}}^{2}dF(z)$; and

5 δ represents E[Y∣X,Z,U].

**Table 2.** Estimates of β in the semiparametric logistic regression for evaluating the risk factors of myocardial ischemia.

	Naive			IPW			AIPW		
Risk Factors	$\hat{\beta }$	SE	p-Value	$\hat{\beta }$	SE	p-Value	$\hat{\beta }$	SE	p-Value
female	−1.56	0.76	0.040	−1.55	0.76	0.043	−1.54	0.76	0.044
smoking	0.36	0.27	0.173	0.51	0.27	0.059	0.51	0.27	0.060
chest pain	0.32	0.49	0.514	0.39	0.49	0.430	0.39	0.49	0.433
blood pressure med.	1.14	0.34	0.001	1.28	0.35	<0.001	1.28	0.35	<0.001
cholesterol med.	1.13	0.84	0.177	1.21	0.84	0.152	1.22	0.84	0.148

## Data Availability

Available upon request.
