# Supplementary material for: Doubly Robust Estimation and Semiparametric Efficiency in Generalized Partially Linear Models with Missing Outcomes
Source: Stats (Basel). Author manuscript; Available in PMC 2025 Sep 30. (PMC12478555; doi:10.3390/stats7030056)
Supplement: supplementary materials [file NIHMS2111172-supplement-supplementary_materials.pdf]

Doubly Robust Estimation and Semiparametric Efficiency in Generalized Partially Linear Models With Missing Outcomes

Lu Wang[1], Zhongzhe Ouyang[1], and Xihong Lin[2]

[1] *Department of Biostatistics, University of Michigan*
[2] *Department of Biostatistics, Harvard School of Public Health*

Throughout this appendix, we assume that $f_Z(\cdot)$, the density of Z, has compact support and z is an interior point of the support. Furthermore, we assume that $f_Z(\cdot)$ is bounded away from 0 on its support and $\pi(\mathcal{X}, Z, \mathcal{U}) > c > 0$ with prob. 1 for some c. In addition, we assume equivalent convexity conditions given by Carroll, Fan, Gijbels, and Wand (1997), which ensure that the kernel–profile estimating equations (5) and (6) have a unique solution $\widehat{\theta}(z)$ and β, which lies in a compact set. We also assume the conditions equivalent to their condition 2 hold. The purpose behind these assumptions is to establish the uniform convergence of $\widehat{\theta}(\cdot)$ and $\widehat{\varphi}$. In what follows, we let $n \to \infty$, $h \to 0$, and $nh \to \infty$.

S1. Semiparametric Efficient Score

We first show that $\Lambda_{nuis}^{\perp} = \{b(\mathcal{X}, Z)\epsilon^* : b(\mathcal{X}, Z) \text{ satisfies } (11)\} \cap L_2(F_O)$. To do so, it suffices to show that $\Lambda_{nuis}^{\perp, full} = \{b(\mathcal{X}, Z)\epsilon : b(\mathcal{X}, Z) \text{ satisfies } (11)\} \cap L_2(F_W)$. The tangent space Λ_{nuis}^{full} is the close linear span of $\Lambda_1^{full} + \Lambda_2^{full}$, where Λ_1^{full} is the linear span of nuisance scores in submodel $\mathcal{A}_{1,full}$ of $\mathcal{A}_{full}$ in which $\theta(\cdot) = \theta_0(\cdot)$ is fixed and known, and Λ_2^{full} is the linear span of scores in the submodel $\mathcal{A}_{2,full}$ of $\mathcal{A}_{full}$ in which the law of W is known up to $\theta(\cdot)$. Thus, $\Lambda_{nuis}^{\perp, full} = \Lambda_1^{\perp, full} \cap \Lambda_2^{\perp, full}$. Because under $\mathcal{A}_{1,full}$, $\theta(\cdot)$ is known, then $\mathcal{A}_{1,full}$ is defined just by a parametric restriction on the conditional mean of Y given $(\mathcal{X}, Z)$. The orthocomplement

of the nuisance tangent space in such a model is

$$\Lambda_1^{\perp,full} = \{b(\mathcal{X},Z)\epsilon : \ b(\mathcal{X},Z) \text{ is an arbitrary function of } (\mathcal{X},Z)\} \cap L_2(F_W) \tag{S1}$$

(see, for example, van der Laan and Robins, 2003, Lemma 2.1). On the other hand, to derive $\Lambda_2^{\perp,full}$, consider a regular parametric submodel $t \to F_{W,t}$ for the law of W under model $\mathcal{A}_{2,full}$ with $F_{W,t=0} = F_W$ and let S_{par} be its score at $t = 0$. Such a parametric submodel is determined by a parametric submodel $t \to \theta(\cdot;t)$ for $\theta(\cdot)$ with $\theta(\cdot;t)$ differentiable with respect to t. Because any parametric submodel of $\mathcal{A}_{2,full}$ only restricts the law of Y given $(\mathcal{X},Z)$, it follows that

$$E(S_{par}|\mathcal{X},Z) = 0 \tag{S2}$$

where throughout, $E(\cdot)$ stands for expectation computed under F_W. If $E_t(\cdot)$ denotes expectation with respect to $F_{W,t}$, then $\partial E_t(Y|\mathcal{X},Z)/\partial t|_{t=0} = E(YS_{par}|\mathcal{X},Z)$ (Ibragimov and Hasminskii, 1981, Lemma 7.2). Thus, taking derivatives with respect to t on both sides of $E_t(Y|\mathcal{X},Z) = \mu\left(\mathcal{X}^T\beta_0 + \theta(Z;t)\right)$ and using that by $(S2)$, $E(YS_{par}|\mathcal{X},Z) = E(\epsilon S_{par}|\mathcal{X},Z)$, we obtain that S_{par} must satisfy

$$E(\epsilon S_{par}|\mathcal{X},Z) = u(Z)\mu^{(1)} \tag{S3}$$

where $u(X) = \partial\theta(Z;t)/\partial t|_{t=0}$ and $\mu^{(1)} \equiv \mu^{(1)}\left(\mathcal{X}^T\beta_0 + \theta_0(Z)\right)$. We thus conclude that Λ_2^{full} is included in the set

$$\left\{S = s(W) : E(S|\mathcal{X},Z) = 0 \text{ and } E(\epsilon S|\mathcal{X},Z) = u(Z)\mu^{(1)} \text{ for some } u(\cdot)\right\} \cap L_2(F_W) \tag{S4}$$

In fact, Λ_2^{full} is equal to this set because given $u(\cdot)$, the parametric submodel corresponding to $t \to \theta_0(\cdot) + tu(\cdot)$ has a score at $t = 0$ satisfying $(S3)$.

From $\Lambda_{nuis}^{\perp,full} = \Lambda_1^{\perp,full} \cap \Lambda_2^{\perp,full}$ and the equality $(S1)$, we conclude that $\Lambda_{nuis}^{\perp,full}$ comprises random vectors of the form $b(\mathcal{X},Z)\epsilon \in L_2(F_W)$ such that

$$E[b(\mathcal{X},Z)\epsilon S] = 0 \text{ for all } S \text{ in the set (S4)}$$

But $E\left[b(\mathcal{X},Z)E\left(\epsilon S|\mathcal{X},Z\right)\right]=0$ for all S in (S4) if and only if $E\left[b(\mathcal{X},Z)\mu^{(1)}u\left(Z\right)\right]=0$ for all $u\left(Z\right)$ or, equivalently, if and only if (11) holds, which proves the claim made about the set $\Lambda_{nuis}^{\perp,full}$.

We now derive $S_{\beta,eff}$, the semiparametric efficient score for β in model $\mathcal{A}$. Noting that

$$\left\{b(\mathcal{X},Z):E[b(\mathcal{X},Z)\mu^{(1)}|Z]=0\right\}=\left\{b(\mathcal{X},Z)-\frac{E[b(\mathcal{X},Z)\mu^{(1)}|Z]}{E[\mu^{(1)}|Z]}:b(\mathcal{X},Z)\text{ arbitrary}\right\}$$

we conclude from (10) that

$$\Lambda_{nuis}^{\perp}=\left\{\left(b(\mathcal{X},Z)-E[b(\mathcal{X},Z)\mu^{(1)}|Z]/E[\mu^{(1)}|Z]\right)\epsilon^{*}:b(\mathcal{X},Z)\text{ arbitrary}\right\}\cap L_{2}\left(F_{O}\right),$$

Let $\mathcal{S}_{\beta}$ be the score for β in the parametric submodel $\mathcal{A}_{fix}$ of $\mathcal{A}$ in which only β is unknown. By definition, $\mathcal{S}_{\beta,eff}$ is the unique $p\times1$ vector with components in $\Lambda_{nuis}^{\perp}$ that satisfies that $\mathcal{S}_{\beta}-\mathcal{S}_{\beta,eff}$ is uncorrelated with all the members of $\Lambda_{nuis}^{\perp}$. Thus,

$$E\left[\left(\mathcal{S}_{\beta}-\mathcal{S}_{\beta,eff}\right)\epsilon^{*}\left\{b(\mathcal{X},Z)-E[b(\mathcal{X},Z)\mu^{(1)}|Z]/E[\mu^{(1)}|Z]\right\}\right]=0,\ \text{for all } b(\mathcal{X},Z) \tag{S5}$$

or equivalently, letting $\mathcal{M}\equiv\left(S_{\beta}-\mathcal{S}_{\beta,eff}\right)\epsilon^{*}$,

$$\begin{aligned}E\left[\mathcal{M}b(\mathcal{X},Z)\right] &= E\{\mathcal{M}E[b(\mathcal{X},Z)\mu^{(1)}|Z]/E[\mu^{(1)}|Z]\}\\ &= E\left\{E\left(\mathcal{M}|Z\right)b(\mathcal{X},Z)\mu^{(1)}/E[\mu^{(1)}|Z]\right\}\ \text{for all } b(\mathcal{X},Z)\end{aligned}$$

The last display is equivalent to $E\left[\mathcal{M}-E\left(\mathcal{M}|Z\right)\mu^{(1)}/E\left(\mu^{(1)}|Z\right)\middle|\mathcal{X},Z\right]=0$, which, in turn, is equivalent to

$$E\left(\mathcal{M}|\mathcal{X},Z\right)=E\left(\mathcal{M}|Z\right)\mu^{(1)}/E\left(\mu^{(1)}|Z\right). \tag{S6}$$

Now

$$E\left(\mathcal{M}|\mathcal{X},Z\right)=E\left(\mathcal{S}_{\beta}\epsilon^{*}|\mathcal{X},Z\right)-E\left(\mathcal{S}_{\beta,eff}\epsilon^{*}|\mathcal{X},Z\right) \tag{S7}$$

and it follows from Ibragimov and Hasminskii (Lemma 7.2) and simple algebra that

$$E\left[\mathcal{S}_{\beta}\epsilon^{*}|\mathcal{X},Z\right]=\frac{\partial E_{\beta}\left(\epsilon^{*}|\mathcal{X},Z\right)}{\partial\beta}=-\mathcal{X}\mu^{(1)} \tag{S8}$$

where $E_\beta(\cdot)$ denotes expectation under the law in submodel $\mathcal{A}_{fix}$ corresponding to the specific value β. Writing $\mathcal{S}_{\beta,eff} = b_{eff}(\mathcal{X}, Z)\epsilon^*$ for some $b_{eff}(\mathcal{X}, Z)$ satisfying $E[b_{eff}(\mathcal{X}, Z)\mu^{(1)}|Z] = 0$, we obtain from $(S6)$, $(S7)$, and $(S8)$ and some algebra that

$$b_{eff}(\mathcal{X}, Z) = -\sigma^{-2}(\mathcal{X}, Z)\mu^{(1)} \times \left\{\mathcal{X} + E\left(\mathcal{M}|Z\right)/E\left(\mu^{(1)}|Z\right)\right\} \tag{S9}$$

where $\sigma^2(\mathcal{X}, Z) = E\left[(\epsilon^*)^2|\mathcal{X}, Z\right] = var(\epsilon^*|\mathcal{X}, Z)$. But since $E[b_{eff}(\mathcal{X}, Z)\mu^{(1)}|Z] = 0$, we have that $E\left[\sigma^{-2}(\mathcal{X}, Z)\left(\mu^{(1)}\right)^2 \times \left\{\mathcal{X} + E\left(\mathcal{M}|Z\right)/E\left(\mu^{(1)}|Z\right)\right\}\Big| Z\right] = 0$. Solving this last equality for $E\left(\mathcal{M}|Z\right)/E\left(\mu^{(1)}|Z\right)$, we obtain

$$E\left(\mathcal{M}|Z\right)/E\left(\mu^{(1)}|Z\right) = -\ E\left[\sigma^{-2}(\mathcal{X}, Z)(\mu^{(1)})^2\mathcal{X}|Z\right]\Big/ E\left[\sigma^{-2}(\mathcal{X}, Z)(\mu^{(1)})^2|Z\right]$$

Replacing this expression in the right-hand side of $(S9)$, we arrive at the desired expression.

S2. Proof of Lemma 1 and Lemma 2

Let $\widehat{\theta}_{AIPW}(z, \beta)$ be the solution of the AIPW kernel estimating equation (5) for a given β. Let $\theta_{AIPW}(z, \beta) = P\lim_{n\to\infty}\widehat{\theta}_{AIPW}(z, \beta)$, $\varphi_{AIPW}(z, \beta) = \partial\theta_{AIPW}(z,\ \beta)/\partial\beta$, $\mu_i(z, \beta, \alpha) \equiv \mu\{\mathcal{X}_i^T\beta + \mathcal{G}(Z_i - z)^T\alpha\}$, $V_i = V[\mu\{\mathcal{X}_i^T\beta + \theta(Z_i)\}; \zeta^*]$, where $\zeta^* = \mathrm{P}\lim_{n\to\infty}\widehat{\zeta}$ and $\widetilde{\pi}(\mathcal{X}, Z, \mathcal{U})$, and let $\widetilde{\delta}(\mathcal{X}, Z, \mathcal{U})$ be defined as in Theorem 1. Then $\theta_{AIPW}(z, \beta) = \alpha_{AIPW,0}$, where $\alpha_{AIPW} \equiv (\alpha_{AIPW,0}, \alpha_{AIPW,1})$, satisfies

$$\mathrm{P}\lim_{n\to\infty}\frac{1}{n}\sum_{i=1}^{n} K_h(Z_i - z)\mu_i^{(1)}(z, \beta, \alpha_{AIPW})V_i^{-1}\mathcal{G}(Z_i - z)\left\{\frac{R_i}{\widetilde{\pi}(\mathcal{X}_i, Z_i, \mathcal{U}_i)}\left[Y_i - \mu_i(z, \beta, \alpha_{AIPW})\right]\right.$$
$$\left. -(\frac{R_i}{\widetilde{\pi}(\mathcal{X}_i, Z_i, \mathcal{U}_i)} - 1)\left[\widetilde{\delta}(\mathcal{X}_i, Z_i, \mathcal{U}_i) - \mu_i(z, \beta, \alpha_{AIPW})\right]\right\} = 0$$

Taking the derivative with respect to β and evaluating at β_0 on both sides, we have

$$\begin{aligned}
&\mathrm{P}\lim_{n\to\infty}\frac{1}{n}\sum_{i=1}^{n}K_h(Z_i-z)\mathcal{G}(Z_i-z)\left\{\frac{R_i}{\widetilde{\pi}(\mathcal{X}_i,Z_i,\mathcal{U}_i)}\frac{\partial\mu_i^{(1)}(z,\beta_0,\alpha_{AIPW})V_i^{-1}}{\partial\beta}\left[Y_i-\mu_i\left(z,\beta_0,\alpha_{AIPW}\right)\right]\right.\\
&-\frac{R_i}{\widetilde{\pi}(\mathcal{X}_i,Z_i,\mathcal{U}_i)}\left\{\mu_i^{(1)}(z,\beta_0,\alpha_{AIPW})\right\}^2V_i^{-1}\left[\varphi_{AIPW}(z,\beta_0)+\mathcal{X}_i^T\right]\\
&-\left\{\frac{R_i}{\widetilde{\pi}(\mathcal{X}_i,Z_i,\mathcal{U}_i)}-1\right\}\frac{\partial\mu_i^{(1)}(z,\beta_0,\alpha_{AIPW})V_i^{-1}}{\partial\beta}\left\{\widetilde{\delta}(\mathcal{X}_i,Z_i,\mathcal{U}_i)-\mu_i(z,\beta_0,\alpha_{AIPW})\right\}\\
&\left.+\left\{\frac{R_i}{\widetilde{\pi}(\mathcal{X}_i,Z_i,\mathcal{U}_i)}-1\right\}\left\{\mu_i^{(1)}(z,\beta_0,\alpha_{AIPW})\right\}^2V_i^{-1}\left[\varphi_{AIPW}(z,\beta_0)+\mathcal{X}_i^T\right]\right\}=0.
\end{aligned}$$

The sum of the first and third term evaluated at β_0 is equal to 0 if π_i is either equal to or a consistent estimate of $P\left(R_i=1|\mathcal{X}_i,Z_i,\mathcal{U}_i\right)$ or if $\delta(\mathcal{X}_i,Z_i,\mathcal{U}_i)=E(Y_i|\mathcal{X}_i,Z_i,\mathcal{U}_i)$ or consistent estimates of it. Therefore, we have

$$\mathrm{P}\lim_{n\to\infty}\frac{1}{n}\sum_{i=1}^{n}K_h(Z_i-z)\{\mu_i^{(1)}(z,\beta_0,\alpha_{AIPW})\}^2V_i^{-1}\mathcal{G}(Z_i-z)[\varphi_{AIPW}(z,\beta_0)+\mathcal{X}_i^T]=0. \quad \text{(S10)}$$

Furthermore, it can be shown that

$$\begin{aligned}
&\mathrm{P}\lim_{n\to\infty}\frac{1}{n}\sum_{i=1}^{n}K_h(Z_i-z)\{\mu_i^{(1)}(z,\beta_0,\alpha_{AIPW})\}^2V_i^{-1}\varphi_{AIPW}(z,\beta_0)\\
=\;& f_Z(z)\varphi_{AIPW}(z,\beta_0)E\left[\{\mu^{(1)}(Z,\beta_0,\alpha_{AIPW})\}^2V^{-1}|Z=z\right], \quad \text{(S11)}
\end{aligned}$$

and

$$\begin{aligned}
&\mathrm{P}\lim_{n\to\infty}\frac{1}{n}\sum_{i=1}^{n}K_h(Z_i-z)\{\mu_i^{(1)}(z,\beta_0,\alpha_{AIPW})\}^2V_i^{-1}\mathcal{X}_i^T\\
=\;& f_Z(z)E\left[\{\mu^{(1)}(Z,\beta_0,\alpha_{AIPW})\}^2V^{-1}\mathcal{X}^T|Z=z\right]. \quad \text{(S12)}
\end{aligned}$$

Lemma 1 follows immediately from (S10), (S11), and (S12).

Likewise, we can prove Lemma 2. Let $\widehat{\theta}_{IPW}(z,\beta)$ denote the solution of the IPW kernel estimating equation (7) for any given β, $\theta_{IPW}(z,\beta)$ $=\mathrm{P}\lim_{n\to\infty}\widehat{\theta}_{IPW}(z,\beta)$ and $\varphi_{IPW}(z,\beta)=\partial\theta_{IPW}(z,\beta)/\partial\beta$. Then $\theta_{IPW}(z,\beta)=\alpha_{IPW,0}$, where $\alpha_{IPW}\equiv(\alpha_{IPW,0},\alpha_{IPW,1})$, satisfies

$$\mathrm{P}\lim_{n\to\infty}\frac{1}{n}\sum_{i=1}^{n}\frac{R_i}{\widetilde{\pi}(\mathcal{X}_i,Z_i,\mathcal{U}_i)}K_h(Z_i-z)\mu_i^{(1)}(z,\beta,\alpha_{IPW})V_i^{-1}\mathcal{G}(Z_i-z)[Y_i-\mu_i(z,\beta,\alpha_{IPW})]=0.$$

Taking the derivative with respect to β and evaluating at β_0 on both sides, we obtain

$$\begin{aligned} & \mathrm{P}\lim_{n\to\infty} \frac{1}{n}\sum_{i=1}^{n}\left[\frac{R_i K_h(Z_i-z)}{\widetilde{\pi}(\mathcal{X}_i,Z_i,\mathcal{U}_i)}\mathcal{G}(Z_i-z)\frac{\partial\mu_i^{(1)}(z,\beta_0,\alpha_{IPW})}{\partial\beta}V_i^{-1}\left\{Y_i-\mu_i(z,\beta_0,\alpha_{IPW})\right\}\right] \\ = \ & \mathrm{P}\lim_{n\to\infty} \frac{1}{n}\sum_{i=1}^{n}\left[\frac{R_i K_h(Z_i-z)}{\widetilde{\pi}(\mathcal{X}_i,Z_i,\mathcal{U}_i)}\{\mu_i^{(1)}(z,\beta_0,\alpha_{IPW})\}^2 V_i^{-1}\mathcal{G}(Z_i-z)\left\{\varphi_{IPW}(z,\beta_0)+\mathcal{X}_i^T\right\}\right] \end{aligned}$$

If the IPW equation uses either the true selection probabilities or their consistent estimators, then, consequently, the left-hand side in the preceding display has Plim equal to 0, and Lemma 2 follows after a few steps of algebra, similar toderiving (S10), (S11), and (S12).

S3. Proof of Theorem 3 and Corollary 2

Let $\mathcal{D}_i(\beta,\zeta)$ denote $\widetilde{\mu}_i^{(1)}(\beta)V_i^{-1}\{\mathcal{X}_i+\partial\widehat{\theta}(Z_i,\beta)/\partial\beta\}$. To simplify notation, we write $\widehat{\beta} = \widehat{\beta}_{AIPW,\delta}$ and $\widehat{\theta}(\cdot)=\widehat{\theta}_{AIPW,\delta}\left(\cdot,\widehat{\beta}_{AIPW,\delta}\right)$ for short. We first note that the consistency of $\widehat{\beta}$ and $\widehat{\theta}(\cdot)$ when either model (3) or model (9) is correct follows from the fact that $E\left[\epsilon_i\left(\tau_0,\eta^*,\beta_0,\theta\right)|\mathcal{X}_i,Z_i\right]=0$ if either model (3) is correct or model (9) is correct. To prove Theorem 3, we use the fact that $n^{-1/2}\sum_{i=1}^{n}\mathcal{D}_i(\widehat{\beta},\widehat{\zeta})\epsilon_i(\widehat{\tau},\widehat{\eta},\widehat{\beta},\widehat{\theta})$ and $n^{-1/2}\sum_{i=1}^{n}\mathcal{D}_i(\widehat{\beta},\zeta^*)\epsilon_i(\widehat{\tau},\widehat{\eta},\widehat{\beta},\widehat{\theta})$ differ by $o_p(1)$, and we write

$$\begin{aligned} 0 \ &= \ \frac{1}{\sqrt{n}}\sum_{i=1}^{n}\mathcal{D}_i\left(\widehat{\beta},\zeta^*\right)\left\{\epsilon_i\left(\widehat{\tau},\widehat{\eta},\widehat{\beta},\widehat{\theta}\right)-\epsilon_i\left(\widehat{\tau},\widehat{\eta},\widehat{\beta},\theta\right)\right\}+\frac{1}{\sqrt{n}}\sum_{i=1}^{n}\mathcal{D}_i\left(\widehat{\beta},\zeta^*\right)\epsilon_i\left(\widehat{\tau},\widehat{\eta},\widehat{\beta},\theta\right)+o_p(1) \\ &= \ \frac{1}{\sqrt{n}}\sum_{i=1}^{n}\mathcal{D}_i\left(\widehat{\beta},\zeta^*\right)\left\{\epsilon_i\left(\widehat{\tau},\widehat{\eta},\widehat{\beta},\widehat{\theta}\right)-\epsilon_i\left(\widehat{\tau},\widehat{\eta},\widehat{\beta},\theta\right)\right\}+\frac{1}{\sqrt{n}}\sum_{i=1}^{n}\mathcal{D}_i\left(\beta_0,\zeta^*\right)\epsilon_i\left(\tau^*,\eta^*,\beta_0,\theta\right) \\ &+ \ \frac{1}{n}\frac{\partial}{\partial\beta^T}\left\{\sum_{i=1}^{n}\mathcal{D}_i\left(\beta_0,\zeta^*\right)\epsilon_i\left(\tau^*,\eta^*,\beta_0,\theta\right)\right\}\sqrt{n}\left(\widehat{\beta}-\beta_0\right) \\ &+ \ \frac{1}{n}\frac{\partial}{\partial\eta^T}\left\{\sum_{i=1}^{n}\mathcal{D}_i\left(\beta_0,\zeta^*\right)\epsilon_i\left(\tau^*,\eta^*,\beta_0,\theta\right)\right\}\sqrt{n}\left(\widehat{\eta}-\eta^*\right) \\ &+ \ \frac{1}{n}\frac{\partial}{\partial\tau^T}\left\{\sum_{i=1}^{n}\mathcal{D}_i\left(\beta_0,\zeta^*\right)\epsilon_i\left(\tau^*,\eta^*,\beta_0,\theta\right)\right\}\sqrt{n}\left(\widehat{\tau}-\tau^*\right)+o_p(1) \end{aligned}$$

It follows that

$$
\begin{aligned}
0 &= \frac{1}{\sqrt{n}}\sum_{i=1}^{n}\mathcal{D}_i\left(\widehat{\beta},\zeta^*\right)\left\{\epsilon_i\left(\widehat{\tau},\widehat{\eta},\widehat{\beta},\widehat{\theta}\right)-\epsilon_i\left(\widehat{\tau},\widehat{\eta},\widehat{\beta},\theta\right)\right\}+\frac{1}{\sqrt{n}}\sum_{i=1}^{n}\mathcal{D}_i^*\epsilon_i\left(\tau^*,\eta^*,\beta_0,\theta\right)\\
&+ E\left\{\left[\mu^{(1)}\left\{\mathcal{X}^T\beta_0+\theta\left(Z_i\right)\right\}\right]^2 V\left[\mu\left\{\mathcal{X}^T\beta_0+\theta\left(Z\right)\right\};\zeta^*\right]^{-1}\widetilde{\mathcal{X}}\widetilde{\mathcal{X}}^T\right\}\sqrt{n}\left(\widehat{\beta}-\beta_0\right)\\
&- E\left[\mathcal{D}^*\frac{\partial}{\partial\eta^T}\epsilon\left(\tau^*,\eta^*,\beta_0,\theta\right)\right]E\left[\frac{\partial}{\partial\eta^T}l\left(Y,\mathcal{X},\mathcal{Z},\mathcal{U};\eta^*\right)\right]^{-1}\frac{1}{\sqrt{n}}\sum_{i=1}^{n}l\left(Y_i,\mathcal{X}_i,\mathcal{Z}_i,\mathcal{U}_i;\eta^*\right)\\
&- E\left[\mathcal{D}^*\frac{\partial}{\partial\tau^T}\epsilon\left(\tau^*,\eta^*,\beta_0,\theta\right)\right]E\left[\frac{\partial}{\partial\tau^T}\mathcal{S}(R,\mathcal{X},Z,\mathcal{U};\tau^*)\right]^{-1}\frac{1}{\sqrt{n}}\sum_{i=1}^{n}\mathcal{S}_{\tau,i}+o_p\left(1\right) \qquad \text{(S13)}
\end{aligned}
$$

where $\mathcal{S}_{\tau,i}=\partial\log[\pi(\mathcal{X}_i,Z_i,\mathcal{U}_i;\tau)^{R_i}\{1-\pi(\mathcal{X}_i,Z_i,\mathcal{U}_i;\tau)\}^{1-R_i}]/\partial\tau\big|_{\tau^*}$ is the score for τ evaluated at τ^*. As we argue below,

$$
\frac{1}{\sqrt{n}}\sum_{i=1}^{n}\mathcal{D}_i\left(\widehat{\beta},\zeta^*\right)\left\{\epsilon_i\left(\widehat{\tau},\widehat{\eta},\widehat{\beta},\widehat{\theta}\right)-\epsilon_i\left(\widehat{\tau},\widehat{\eta},\widehat{\beta},\theta\right)\right\}=o_p\left(1\right) \qquad \text{(S14)}
$$

Therefore, (S13) implies that $\sqrt{n}\left\{\widehat{\beta}-\beta_0\right\}=-\mathcal{A}\left(V\right)^{-1}\times\frac{1}{\sqrt{n}}\sum_{i=1}^{n}$

$$
\begin{aligned}
&\left\{\mathcal{D}_i^*\epsilon_i\left(\tau^*,\eta^*,\beta_0,\theta\right)-E\left[\mathcal{D}^*\frac{\partial}{\partial\tau^T}\epsilon\left(\tau^*,\eta^*,\beta_0,\theta\right)\right]E\left[\frac{\partial}{\partial\tau^T}\mathcal{S}(R,\mathcal{X},Z,\mathcal{U};\tau^*)\right]^{-1}\mathcal{S}_{\tau,i}\right.\\
&\left.-E\left[\mathcal{D}^*\frac{\partial}{\partial\eta^T}\epsilon\left(\tau^*,\eta^*,\beta_0,\theta\right)\right]E\left[\frac{\partial}{\partial\eta^T}l\left(Y,\mathcal{X},\mathcal{Z},\mathcal{U};\eta^*\right)\right]^{-1}l\left(Y_i,\mathcal{X}_i,\mathcal{Z}_i,\mathcal{U}_i;\eta^*\right)\right\}+o_p\left(1\right),
\end{aligned}
$$

and consequently, by the central limit theorem,

$$
\sqrt{n}\left\{\widehat{\beta}-\beta_0\right\}\to N\left(0,\mathcal{A}\left(V\right)^{-1}\mathcal{B}_\delta\left(V\right)\mathcal{A}\left(V\right)^{-1}\right),
$$

where $\mathcal{A}\left(V\right)$ and $\mathcal{B}_\delta\left(V\right)$ are as defined in Theorem 3. Now it remains to show $(S14)$. Following the mean value theorem, there exist $\overline{\theta}(Z_i,\widehat{\beta})$ such that $|\overline{\theta}(Z_i,\widehat{\beta})-\theta(Z_i)|\leq|\widehat{\theta}(Z_i,\widehat{\beta})-\theta(Z_i)|$ and

$$
\begin{aligned}
&\frac{1}{\sqrt{n}}\sum_{i=1}^{n}\mathcal{D}_i\left(\widehat{\beta},\zeta^*\right)\left\{\epsilon_i\left(\widehat{\tau},\widehat{\eta},\widehat{\beta},\widehat{\theta}\right)-\epsilon_i\left(\widehat{\tau},\widehat{\eta},\widehat{\beta},\theta\right)\right\}\\
=\;&\frac{1}{\sqrt{n}}\sum_{i=1}^{n}\mathcal{D}_i\left(\widehat{\beta},\zeta^*\right)\left[\mu_i\left\{\mathcal{X}_i^T\widehat{\beta}+\theta\left(Z_i\right)\right\}-\mu_i\left\{\mathcal{X}_i^T\widehat{\beta}+\widehat{\theta}\left(Z_i,\widehat{\beta}\right)\right\}\right]\\
=\;&\frac{1}{\sqrt{n}}\sum_{i=1}^{n}\mathcal{D}_i\left(\widehat{\beta},\zeta^*\right)\mu_i^{(1)}\left\{\mathcal{X}_i^T\widehat{\beta}+\overline{\theta}\left(Z_i,\widehat{\beta}\right)\right\}\left\{\theta\left(Z_i\right)-\widehat{\theta}\left(Z_i,\widehat{\beta}\right)\right\}.
\end{aligned}
$$

Under regularity conditions, the last expression is equal to

$$\frac{1}{\sqrt{n}}\sum_{i=1}^{n}\mathcal{D}_i^*\mu_i^{(1)}\left\{\mathcal{X}_i^T\beta_0+\theta\left(Z_i\right)\right\}\left\{\theta\left(Z_i\right)-\widehat{\theta}\left(Z_i,\beta_0\right)\right\}+o_p\left(1\right).$$

Following the same argument as in Wang et al. 2005 Lemma A.1, one can show that the first term is also $o_p(1)$, and therefore $(S14)$ follows. This concludes the proof of Theorem 3.

To prove Corollary 2, first note $\mathcal{A}(V)$ does not depend on δ for a fixed V. So the minimum of $\mathcal{A}(V)^{-1}\mathcal{B}_\delta(V)\mathcal{A}(V)^{-1}$ is achieved at the minimizer of $\mathcal{B}_\delta(V)$. Since $E[\mathcal{D}^*\epsilon_\delta^*(\beta_0,\theta)\mathcal{S}_\tau^T]E[\mathcal{S}_\tau\mathcal{S}_\tau^T]^{-1}\mathcal{S}_\tau$ is the projection of $\mathcal{D}^*\epsilon_\delta^*(\beta_0,\theta)$ on $\mathcal{S}_\tau$, we rewrite it as $d(\mathcal{X},Z,\mathcal{U})\{R-\pi_0(\mathcal{X},Z,\mathcal{U})\}$. Then we can write $\mathcal{D}^*\epsilon_\delta^*(\beta_0,\theta)-E[\mathcal{D}^*\epsilon_\delta^*(\beta_0,\theta)\mathcal{S}_\tau^T]E[\mathcal{S}_\tau\mathcal{S}_\tau^T]^{-1}\mathcal{S}_\tau$ as

$$\mathcal{D}^*\frac{R}{\pi_0\left(\mathcal{X},Z,\mathcal{U}\right)}\cdot\left[Y-\mu\left\{\mathcal{X}^T\beta_0+\theta\left(Z\right)\right\}\right]-j\left(\mathcal{X},Z,\mathcal{U}\right)\left\{R-\pi_0\left(\mathcal{X},Z,\mathcal{U}\right)\right\},$$

where $j(\mathcal{X},Z,\mathcal{U})=d(\mathcal{X},Z,\mathcal{U})+\mathcal{D}^*\pi_0(\mathcal{X},Z,\mathcal{U})^{-1}[\delta(\mathcal{X},Z,\mathcal{U})-\mu\{\mathcal{X}^T\ \beta_0+\theta(Z)\}]$. Denote $\Lambda=\{a(\mathcal{X},Z,\mathcal{U})\{R-\pi_0(\mathcal{X},Z,\mathcal{U})\}:a(\mathcal{X},Z,\mathcal{U})\text{ is arbitrary}\}$, then for $\delta_{opt}(\mathcal{X},Z,\mathcal{U})=E(Y|\mathcal{X},Z,\mathcal{U})$ it can be checked that

$$\begin{aligned}\mathcal{D}^*\epsilon_{\delta_{opt}}^*(\beta_0,\theta) &= \mathcal{D}^*\frac{R}{\pi_0\left(\mathcal{X},Z,\mathcal{U}\right)}\cdot\left(Y-\mu\left\{\mathcal{X}^T\beta_0+\widetilde{\theta}\left(Z\right)\right\}\right)\\ &- \Pi\quad\left[\mathcal{D}^*\frac{R}{\pi_0\left(\mathcal{X},Z,\mathcal{U}\right)}\cdot\left(Y-\mu\left\{\mathcal{X}^T\beta_0+\widetilde{\theta}\left(Z\right)\right\}\right)|\Lambda\right]\end{aligned}$$

where $\Pi[\cdot|\Lambda]$ denotes projection onto Λ in $L_2(P_\mathcal{O})$, and $P_\mathcal{O}$ is the law of the observed data. Consequently, $\mathcal{D}^*\epsilon_{\delta_{opt}}^*(\beta_0,\theta)$ is uncorrelated with the elements of Λ. In particular, $E[\mathcal{D}^*\epsilon_{\delta_{opt}}^*(\beta_0,\theta)\mathcal{S}_\tau^T]=0$ since $\mathcal{S}_\tau$ is in Λ. It follows that $\mathcal{B}_{\delta_{opt}}(V)=var\{\mathcal{D}^*\epsilon_{\delta_{opt}}^*(\beta_0,\theta)\}$, which is the same as $\widetilde{\mathcal{B}}_{\delta_{opt}}(V)$. Thus, we conclude that $\otimes_{\delta_{opt}}(V)=\widetilde{\Omega}_{\delta_{opt}}(V)$. Further, according to the property of projection,

$$\begin{aligned}&var\left(\mathcal{D}^*\frac{R\left[Y-\mu\left\{\mathcal{X}^T\ \beta_0+\theta\left(Z\right)\right\}\right]}{\pi_0\left(\mathcal{X},Z,\mathcal{U}\right)}-\Pi\left[\mathcal{D}^*\frac{R\left[Y-\mu\left\{\mathcal{X}^T\beta_0+\theta\left(Z\right)\right\}\right]}{\pi_0\left(\mathcal{X},Z,\mathcal{U}\right)}|\Lambda\right]\right)\\ \leq\ &var\left(\mathcal{D}^*\frac{R\left[Y-\mu\left\{\mathcal{X}^T\beta_0+\theta\left(Z\right)\right\}\right]}{\pi_0\left(\mathcal{X},Z,\mathcal{U}\right)}-j\left(\mathcal{X},Z,\mathcal{U}\right)\left\{R-\pi_0\left(\mathcal{X},Z,\mathcal{U}\right)\right\}\right)\text{ for any } j.\end{aligned}$$

This ends the proof that $\Omega_{\delta_{opt}}(V)\leq\Omega_\delta(V)$ for any δ. As we argued after Corollary 1, $\mathcal{B}_\delta(V)\leq\widetilde{\mathcal{B}}_\delta(V)$ for any δ whenever model (3) is correct. Thus $\Omega_\delta(V)\leq\widetilde{\Omega}_\delta(V)$ in this

situation, and it follows that $\Omega_{\delta_{opt}}(V) \leq \widetilde{\Omega}_{\delta}(V)$ for any δ. To prove part (iii) of Corollary 2, we note that $\Omega_{\delta_{opt}}(V) = \mathcal{A}(V)\mathcal{B}_{\delta_{opt}}(V)^{-1}\mathcal{A}(V)^T = var\{\Pi[\mathcal{S}_\beta|\mathcal{D}^*(V)\epsilon^*_{\delta_{opt}}(\beta_0,\theta)]\}^{-1}$, where S_β is the score for β in the model in which all parameters other than β are known. Here we write $\mathcal{D}^*(V)$ instead of $\mathcal{D}^*$ to emphasize the dependence of $\mathcal{D}^*$ on V. Now, defining $\Psi = \{\mathcal{D}^*(V)\epsilon^*_{\delta_{opt}}(\beta_0,\theta) : V \text{ is arbitrary}\}$, it can be checked that $\Pi[\mathcal{S}_\beta|\Psi] = \mathcal{D}^*(\sigma^2_{\delta_{opt}})\widetilde{\epsilon}^*_{\delta_{opt}}(\beta_0,\theta)$. Note $\mathcal{D}^*(V)\epsilon^*_{\delta_{opt}}(\beta_0,\theta) \in \Psi$ for any V, then consequently,

$$\Omega_{\delta_{opt}}(V) = var\left\{\Pi\left[\mathcal{S}_\beta|\mathcal{D}^*(V)\,\epsilon^*_{\delta_{opt}}(\beta_0,\theta)\right]\right\}^{-1} \geq var\left\{\Pi\left[\mathcal{S}_\beta|\Psi\right]\right\}^{-1} = \Omega_{\delta_{opt}}\left(\sigma^2_{\delta_{opt}}\right)$$

Furthermore, it can be easily shown that $\mathcal{B}_{\delta_{opt}}(\sigma^2_{\delta_{opt}}) = \mathcal{A}(\sigma^2_{\delta_{opt}})$, which is symmetric. It follows

$$\Omega_{\delta_{opt}}\left(\sigma^2_{\delta_{opt}}\right) = \mathcal{A}\left(\sigma^2_{\delta_{opt}}\right)^{-1}\mathcal{B}_{\delta_{opt}}\left(\sigma^2_{\delta_{opt}}\right)\left\{\mathcal{A}\left(\sigma^2_{\delta_{opt}}\right)^{-1}\right\}^T = \mathcal{A}\left(\sigma^2_{\delta_{opt}}\right)^{-1}.$$

This concludes the proof of Corollary 2.

S4 Other numerical results

We conducted additional simulations to evaluate the performance of the proposed AIPW kernel–profile estimators under different scenarios of missingness and model specifications, providing additional insights into the method's properties and robustness.

For each replication, we generate random samples of (X, Z, U, Y, R), where Z is generated from a Uniform$(-1, 1)$ distribution, X is generated from Norm$\{Z, 1\}$ + Uniform$(-1, 1)$, U is generated from a Uniform$(0, 4)$ + Norm$(X, 0.05^2)$, and the outcome Y is generated from a normal distribution with mean

$$E\left(Y|X, Z, U\right) = X\beta_1 + m(Z) + U\beta_2 \tag{S15}$$

and variance $\sigma^2_{Y|X,Z,U}$, where $\beta_1 = \beta_2 = 1$, $\sigma^2_{Y|X,Z,U} = 1$, and $m(Z) = \sin(2X)$. Note that Z is correlated with X, U, and Y, and U is correlated with X, Z, and Y. We generate R, the selection indicator, according to $\text{logit}\{\pi_i\} = \tau_0 + \tau_1 \cdot (U_i - a_1)I(a_1 < U_i \leq a_2) + \tau_1 \cdot (a_2 - a_1)I(U_i > a_2)$, where $\pi_i = P(R_i = 1|X_i, Z_i, U_i)$ is the probability that subject i is selected to the second stage, $\tau_0 = -1$, $\tau_1 = 1$, $a_1 = 0$, and $a_2 = 3.5$. Based on this selection mechanism, the Monte Carlo median missing percentage of the outcome Y is around 33%. Since the selection probability depends on U only, the assumption of missing at random holds. Note that $E(Y|X,Z) = X\beta + \theta(Z)$, where the true $\beta = 2$ and true $\theta(Z) = m(Z) + 2$. The conclusions from these new simulations are similar to those shown before.

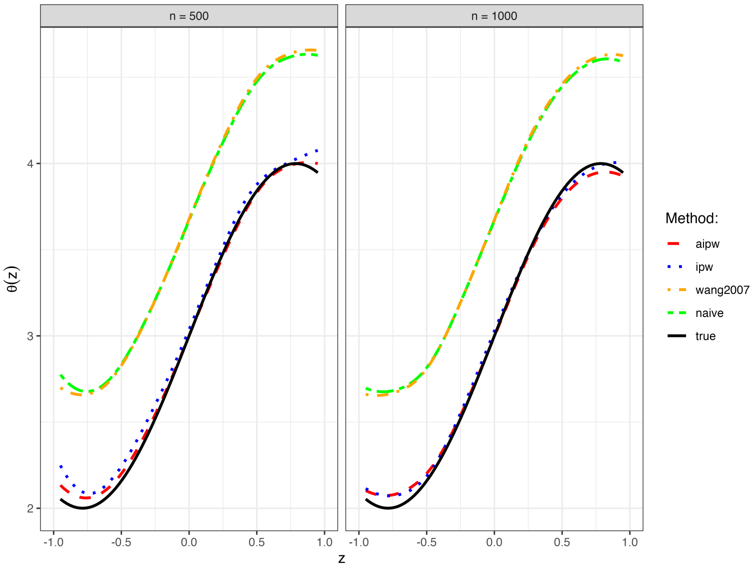

Figure S1: The true $\theta(z)$ and the estimated nonparametric functions $\widehat{\theta}(z)$ through naive, IPW, and AIPW kernel estimating equations based on 100 replications.

Table S1: Simulation results of the naive, IPW, and AIPW kernel–profile estimates based on 100 replications (sample size n=500). Note: 1. Relative bias is defined as $\int |\widehat{bias}\{\widehat{\theta}(z)\}/\theta(z)|dF(z)$; 2. EMP S.E. is the empirical S.E., defined as $\int \widehat{SE}_{EMP}\{\widehat{\theta}(z)\}dF(z)$, where $\widehat{SE}_{EMP}\{\widehat{\theta}(z)\}$ is the sampling S.E. of the replicated $\widehat{\theta}(z)$; 3. EST S.E. is the estimated S.E., defined as $\int \widehat{SE}_{EST}\{\widehat{\theta}(z)\}dF(z)$, where $\widehat{SE}_{EST}\{\widehat{\theta}(z)\}$ is the sampling average of the replicated sandwich estimates $\widehat{SE}\{\widehat{\theta}(z)\}$; 4. EMP MISE is the empirical MISE, defined as $\int\{\widehat{\theta}(z)-\theta(z)\}^2 dF(z)$; and 5. δ represents $E[Y|X,Z,U]$.

	**Kernel Estimator of $\theta(\cdot)$**				**Profile Estimator of β**			
	Relative bias[1]	EMP S.E.[2]	EST S.E.[3]	EMP MISE[4]	Bias of $\widehat{\beta}$	EMP S.E.	EST S.E.	EMP MSE
**Naive Estimator**	0.239	0.260	0.270	0.509	0.118	0.205	0.082	0.079
**IPW Estimator**								
True π	0.094	0.320	0.297	0.110	0.096	0.240	0.225	0.058
Consistent $\widehat{\pi}$	0.092	0.315	0.299	0.107	0.095	0.239	0.150	0.057
Wrong π	0.239	0.261	0.271	0.508	0.116	0.209	0.226	0.078
**AIPW Estimator**								
True π and δ	0.064	0.224	0.220	0.053	0.067	0.167	0.163	0.028
Consistent $\widehat{\pi}$ and consistent $\widehat{\delta}$	0.064	0.224	0.220	0.053	0.067	0.167	0.163	0.028
Wrong π and consistent $\widehat{\delta}$	0.072	0.250	0.229	0.065	0.073	0.187	0.172	0.035
Consistent $\widehat{\pi}$ and wrong $\widehat{\delta}$	0.075	0.256	0.235	0.068	0.076	0.195	0.180	0.038
Both wrong	0.242	0.266	0.254	0.504	0.091	0.213	0.192	0.051
**Wang2007 Estimator**	0.238	0.252		0.506	0.124	0.195	0.163	0.085

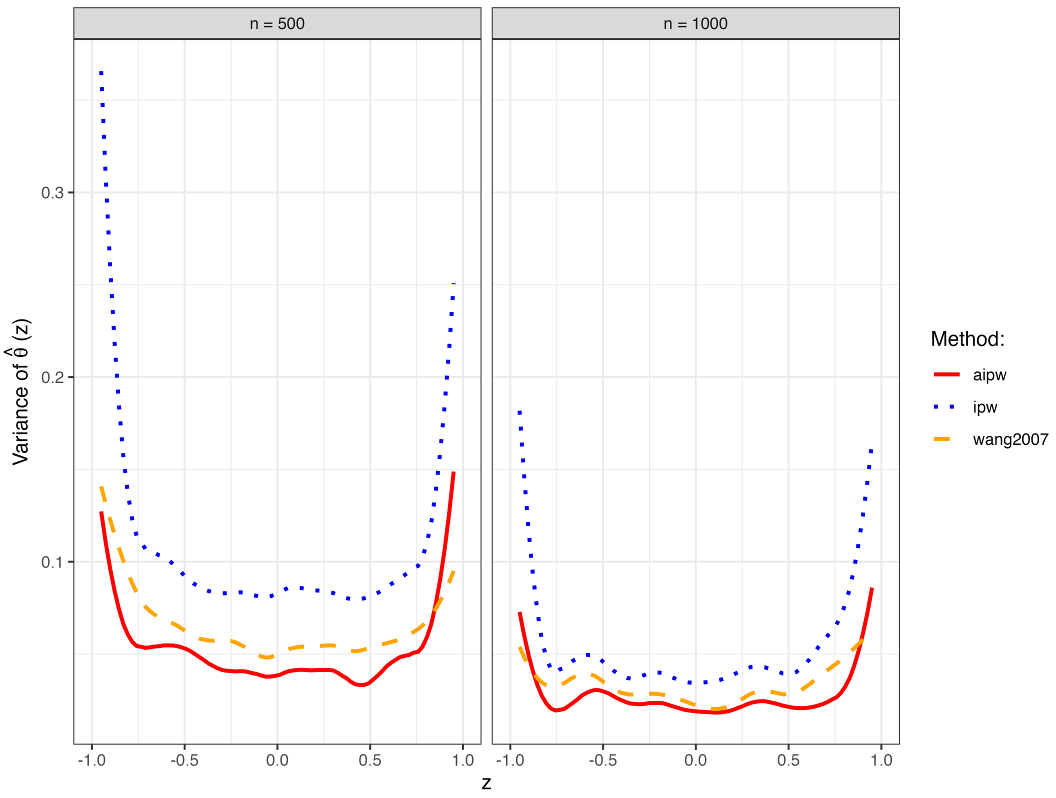

Figure S2: Empirical pointwise variance of the IPW and AIPW estimated nonparametric functions $\widehat{\theta}(z)$ based on 100 replications.

We further compared with the method proposed by Wang and Sun (2007) in simulations and conducted a sensitivity analysis to assess the impact of potential departures from MAR assumptions. In the following, we use wang2007 for short to denote their method. The results show that our method performs very well, while wang2007 has a relatively larger bias and larger variance. Below, we also summarize what we have undertaken regarding sensitivity analysis:

(1) We first conduct a sensitivity analysis for simulation and generate data from a situation

where the missing mechanism is MNAR. We generate R, the selection indicator, according to $\text{logit}\{\pi_i\} = \tau_0 + \tau_1 \cdot (U_i - a_1) I(a_1 < U_i \leq a_2) + \tau_1 \cdot (a_2 - a_1) I(U_i > a_2) + 0.5Y_i$, where $\pi_i = P(R_i = 1 | X_i, Z_i, U_i)$ is the probability that subject i is selected to the second stage, $\tau_0 = -1$, $\tau_1 = 1$, $a_1 = 0$, and $a_2 = 3.5$. Note that here, the missingness depends on Y, which could be missing, and thus the missingness is MNAR. From the simulation results below, we can see that the proposed method outperforms the other methods as it is doubly protected with its double-robustness property.

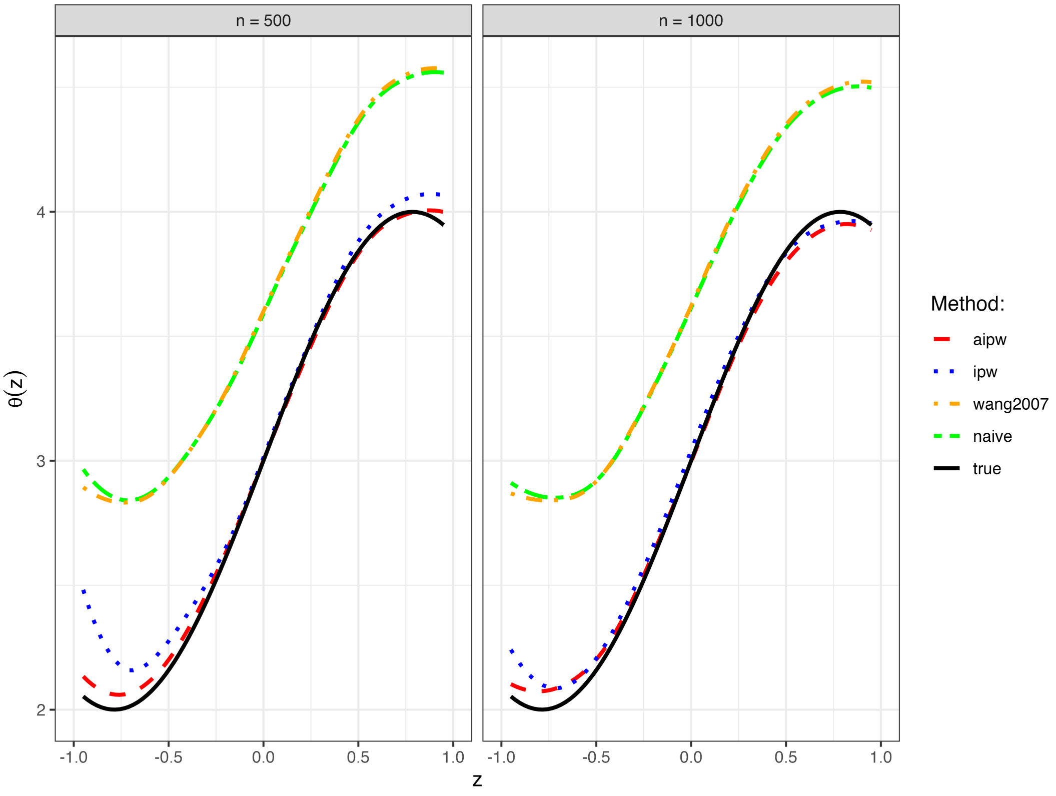

Figure S3: The true $\theta(z)$ and the estimated nonparametric functions $\widehat{\theta}(z)$ through naive, IPW, and AIPW kernel estimating equations based on 100 replications.

Table S2: Simulation results of the naive, IPW, and AIPW kernel–profile estimates based on 100 replications (sample size n=500). Note: 1. Relative bias is defined as $\int |\widehat{bias}\{\widehat{\theta}(z)\}/\theta(z)|dF(z)$; 2. EMP S.E. is the empirical S.E., defined as $\int \widehat{SE}_{EMP}\{\widehat{\theta}(z)\}dF(z)$, where $\widehat{SE}_{EMP}\{\widehat{\theta}(z)\}$ is the sampling S.E. of the replicated $\widehat{\theta}(z)$; 3. EST S.E. is the estimated S.E., defined as $\int \widehat{SE}_{EST}\{\widehat{\theta}(z)\}dF(z)$, where $\widehat{SE}_{EST}\{\widehat{\theta}(z)\}$ is the sampling average of the replicated sandwich estimates $\widehat{SE}\{\widehat{\theta}(z)\}$; 4. EMP MISE is the empirical MISE, defined as $\int \{\widehat{\theta}(z) - \theta(z)\}^2 dF(z)$; and 5. δ represents $E[Y|X, Z, U]$.

	**Kernel Estimator of $\theta(\cdot)$**				**Profile Estimator of β**			
	Relative bias[1]	EMP S.E.[2]	EST S.E.[3]	EMP MISE[4]	Bias of $\widehat{\beta}$	EMP S.E.	EST S.E.	EMP MSE
**Naive Estimator**	0.244	0.233	0.258	0.492	0.191	0.191	0.250	0.180
**IPW Estimator**								
True π	0.107	0.348	0.277	0.140	0.112	0.272	0.256	0.075
Wrong π	0.244	0.234	0.259	0.494	0.197	0.194	0.168	0.190
**AIPW Estimator**								
True π and δ	0.064	0.224	0.220	0.053	0.067	0.167	0.163	0.028
Wrong π and consistent $\widehat{\delta}$	0.073	0.233	0.224	0.072	0.074	0.176	0.165	0.035
Both wrong	0.244	0.237	0.246	0.494	0.136	0.192	0.180	0.102
**Wang2007 Estimator**	0.243	0.225		0.487	0.200	0.184	0.190	0.192

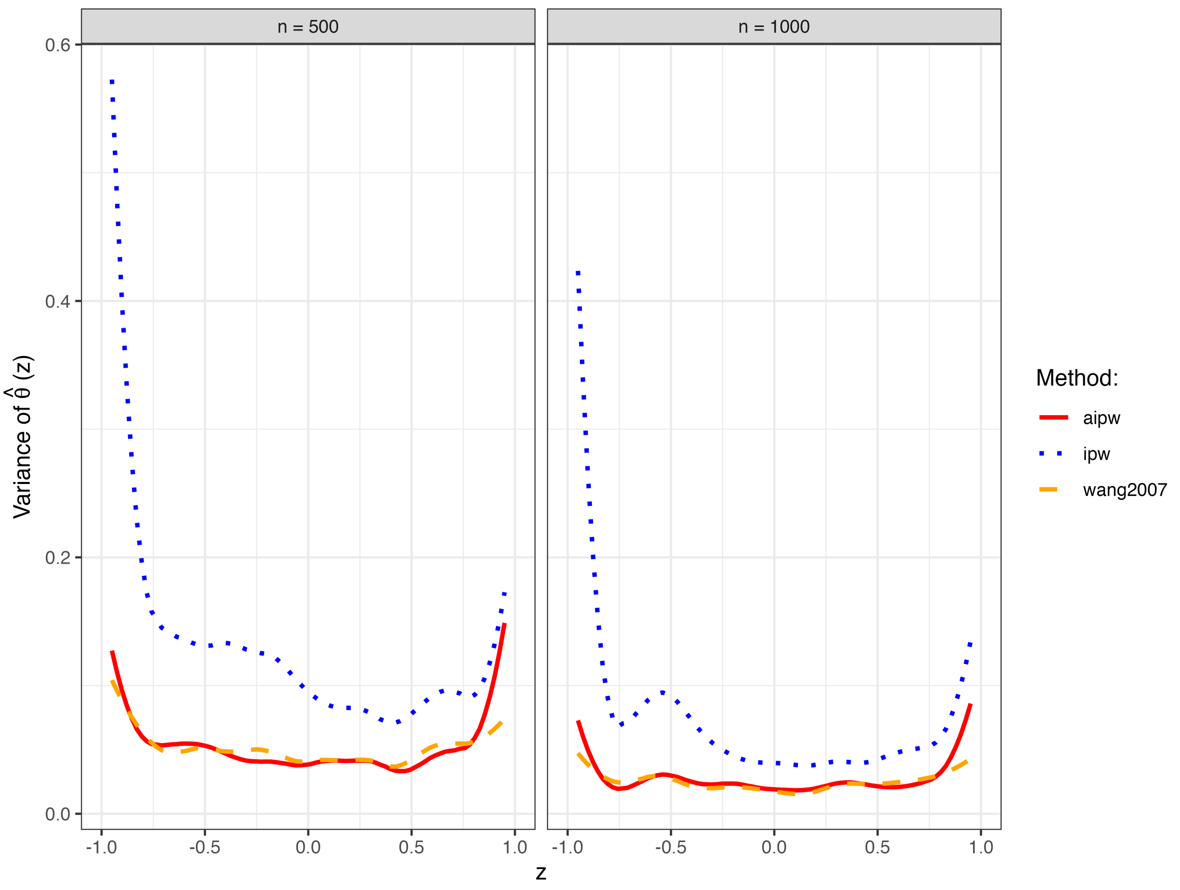

Figure S4: Empirical pointwise variance of the IPW and AIPW estimated nonparametric functions $\widehat{\theta}(z)$ based on 100 replications.

(2) We also conduct sensitivity analysis for the application by first fitting a model of $Pr(R = 1|\mathcal{X}, Z, \mathcal{U}) = \pi(\mathcal{X}, Z, \mathcal{U}; \tau)$ and then adding a sensitivity term with a parameter b to control the level of potential MNAR. For example, with $b = 1$, we allow a moderate level of MNAR, which has 385 observed outcomes with a missing rate of 93.8%. The analysis results are presented as follows, and the AIPW results are not very sensitive in our case. In this study, missingness is

by the protocol design and MAR is quite reasonable.

Table S3: Estimates of β in the semiparametric logistic regression for evaluating the risk factors of myocardial ischemia.

	**Naive**			**IPW**			**AIPW**		
**Risk Factors**	$\widehat{\beta}$	SE	p-value	$\widehat{\beta}$	SE	p-value	$\widehat{\beta}$	SE	p-value
female	-1.35	0.77	0.079	-1.30	0.77	0.092	-1.32	0.77	0.085
smoking	0.45	0.28	0.108	0.62	0.28	0.030	0.57	0.28	0.043
chest pain	0.20	0.54	0.717	0.24	0.54	0.664	0.25	0.54	0.651
blood pressure med.	1.08	0.37	0.004	1.23	0.38	0.001	1.18	0.37	0.002
cholesterol med.	1.24	0.85	0.147	1.35	0.86	0.116	1.30	0.86	0.131

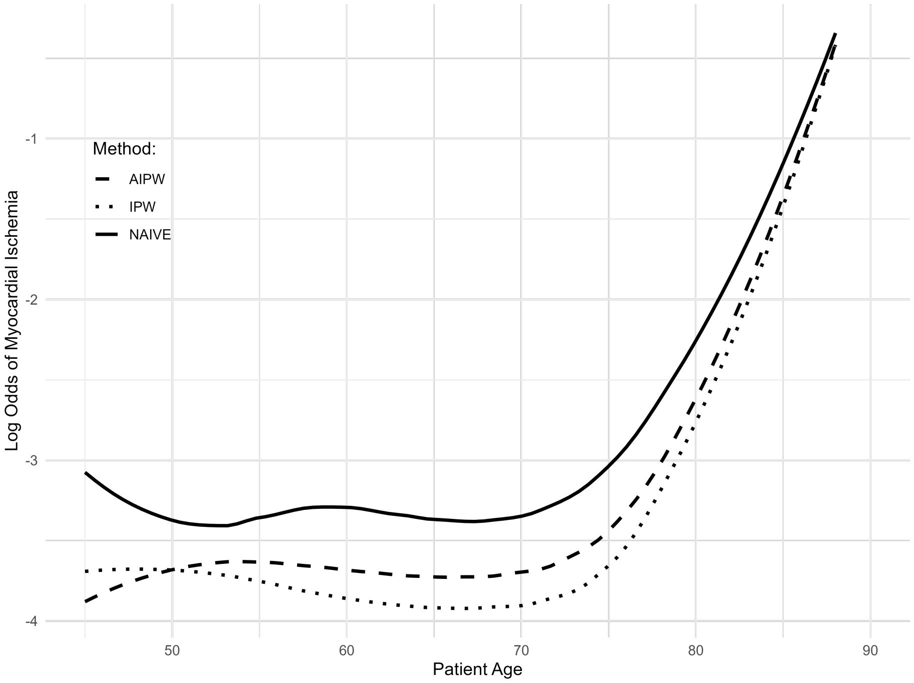

Figure S5: Estimate of $\theta(\text{age})$ for the risk of myocardial ischemia controlled for other potential risk factors and confounders.
